# Lineage Analysis of *Cxcr4*-Expressing Cells in the Developing Midbrain Suggests That Progressive Competence Restriction in Dopaminergic Progenitor Cells Contributes to the Establishment of Dopaminergic Neuronal Diversity

**DOI:** 10.1523/ENEURO.0052-22.2022

**Published:** 2022-08-23

**Authors:** Alessandro Petese, Franca L. Fries, Bianca Broske, Ralf Stumm, Sandra Blaess

**Affiliations:** 1 Neurodevelopmental Genetics, Institute of Reconstructive Neurobiology, Medical Faculty, University of Bonn, 53127 Bonn, Germany; 2 Institute of Pharmacology and Toxicology, Jena University Hospital, 07747 Jena, Germany

**Keywords:** development, dopaminergic diversity, intersectional genetic labeling, lineage tracing, midbrain, mouse

## Abstract

Midbrain dopaminergic (mDA) neurons are generated from a ventral midbrain progenitor zone over a time span of several days [embryonic day 10.0 (E10.0) to E14.5 in mouse]. Within this neurogenic period, a progressively changing fate potential of mDA progenitors could contribute to the generation of diverse mDA neuronal subpopulations. To test this idea, we combined inducible genetic fate mapping and intersectional labeling approaches to trace the lineage of cells expressing the chemokine receptor CXCR4. The *Cxcr4* transcript is expressed in mDA progenitors and precursors, but not in differentiated mDA neurons. *Cxcr4*-expressing mDA progenitors/precursors labeled at E11.5 develop into a broad range of mDA neurons, whereas labeling of the *Cxcr4* lineage at later time points (E12.5–E15.5) results in an increasingly restricted contribution to mDA neurons proceeding from lateral to medial in the substantia nigra and from dorsal to ventral in the ventral tegmental area. In parallel, the innervation of dopaminergic projection targets by mDA neurons derived from *Cxcr4*-expressing cells is becoming more restricted: the late-generated mDA neurons innervate only the medial–rostral regions in the dorsal striatum and only the medial shell in the nucleus accumbens. Our results suggest that mDA progenitor cells become increasingly restricted in their cell fate potential over time.

## Significance Statement

Midbrain dopaminergic (mDA) neurons modulate cognitive processes, voluntary movement, and reward behavior. The degeneration of a subset of mDA neurons results in the motor deficits in Parkinson’s disease, while altered dopamine transmission is associated with neuropsychiatric disorders including depression and schizophrenia. The dopaminergic system is composed of molecularly distinct subpopulations and discrete dopaminergic circuits, which influence distinct aspects of behavior. Understanding how diversity in the dopaminergic system is established is essential for a better insight into its functional output and the mechanisms underlying its dysfunction. Our study shows that mDA progenitors change their competence over time to generate at different developmental time points mDA subtypes that are distinct in their anatomic location and projection targets.

## Introduction

The majority of midbrain dopaminergic (mDA) neurons is located in the substantia nigra pars compacta (SNc), the ventral tegmental area (VTA), and the retrorubral field (RRF). mDA projections to specific targets in the forebrain originate essentially from anatomically distinct populations of mDA neurons: mDA neurons in the medial VTA project to the prefrontal cortex, the amygdala, and medial nucleus accumbens (NAc); dorsolateral VTA–mDA neurons send projections to the lateral nucleus accumbens; mDA neurons in the medial SNc project to the dorsomedial striatum (DMS), and mDA neurons in the lateral SNc to the dorsolateral striatum (DLS; Lammel et al., 2008; Beier et al., 2015; Lerner et al., 2015; Morales and Margolis, 2017; Poulin et al., 2018). Targets of RRF–mDA neurons are not well characterized.

Given the diversity of mDA neurons in the adult brain, the question arises whether and how mDA diversity is established during progenitor specification and differentiation. mDA neurons are generated from progenitors in the floorplate of the ventral midbrain. In this domain, neurogenesis starts around embryonic day 10.0 (E10.0), when the first cells positive for NR4A2 (nuclear receptor 4A2, expressed in mDA precursors and neurons) are detected just below the mDA progenitor zone (Wallén et al., 1999; Dumas and Wallén-Mackenzie, 2019). Shortly after, the cells start to express tyrosine hydroxylase (TH), the rate-limiting enzyme in dopamine synthesis and a marker for mDA neurons. Over the subsequent days of development, mDA neurons continue their differentiation process to develop into mature mDA neurons. Even as the first cohort of mDA precursors and neurons is undergoing this maturation process, neurogenesis (and subsequent maturation steps) from the mDA progenitor domain continues until ∼E14.5 (Bayer et al., 1995; Bye et al., 2012).

**Figure 1.**
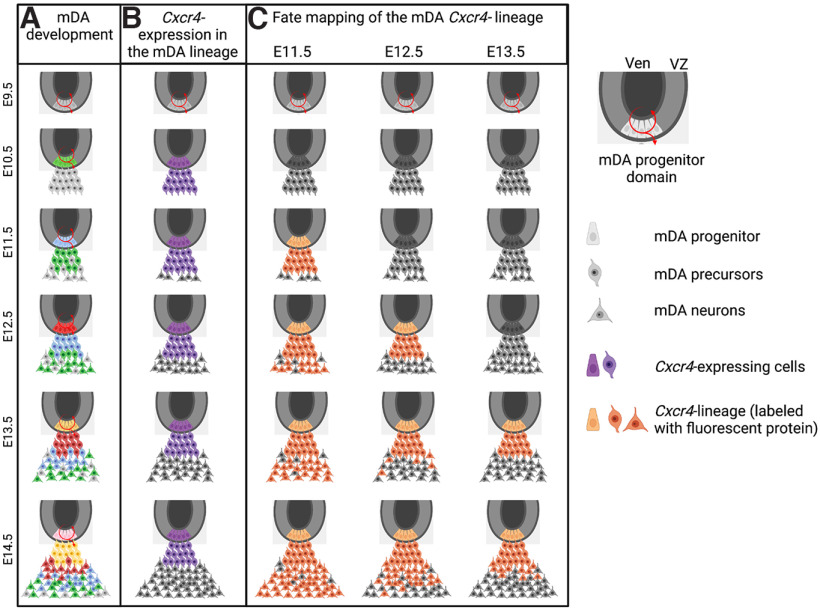
Generation of mDA neurons and *Cxcr4* lineage in the ventral midbrain. ***A***, The mDA progenitor domain is located at the ventral midline of the midbrain and generates mDA neurons between E10.0 and E14.5. Progenitors give rise to mDA precursors (differentiated, but not yet TH expressing) that then mature into mDA neurons. Since neurogenesis occurs over several days, mDA neurons have distinct birthdates, as indicated by the different colors (green, after E10.5; blue, after E11.5; red, after E12.5; yellow, after E13.5; pink, after E14.5). ***B***, *Cxcr4* is expressed in mDA progenitors [and other progenitors in the ventricular zone (VZ), not shown] and in mDA precursors, but is largely absent from mDA neurons (Yang et al., 2013; Bodea et al., 2014). ***C***, Fate-mapping/labeling of *Cxcr4*-expressing mDA progenitors and precursors at different developmental time points allows monitoring of the developmental potential of the progenitors and precursors at specific developmental time points. Ven, Ventricle. This figure was created with https://biorender.com/.

Considering the mechanisms that generate neuronal diversity in other parts of the developing brain, mDA diversity could be generated during progenitor specification and early differentiation through different mechanisms. On one hand, mDA diversity may arise because diverse sets of mDA progenitors are established. In this model, mDA neuronal subclasses are generated from discrete progenitors similar to what has been demonstrated for progenitors of cortical interneurons (Lim et al., 2018). Indeed, two spatially distinct mDA progenitor domains in the ventral midbrain with different molecular profiles have been described: a medial progenitor domain preferentially contributes to SNc–mDA neurons, and a lateral progenitor domain gives rise predominantly to medial VTA neurons (Blaess et al., 2011; Hayes et al., 2011; Panman et al., 2014). While these studies show that some aspects of mDA neuronal diversity are predetermined in mDA progenitors, two distinct progenitor subtypes are not sufficient to account for the functional or molecular diversity of mDA subtypes in the adult brain (Roeper, 2013; Poulin et al., 2020) and as of yet there is no evidence (e.g., from single-cell transcriptomics; Manno et al., 2016) that the two identified mDA progenitor domains are further subdivided. On the other hand, mDA progenitors could change their competence over time to generate different mDA subtypes at different developmental time points. Such a progressive competence restriction is thought to underlie the generation of distinct projection neurons in the cerebral cortex (Llorca and Marín, 2021). It is a plausible model for the formation of neuronal diversity in the dopaminergic system because mDA neurogenesis occurs over several days (E10.0–E14.5 in the mouse), and birth-dating studies indicate that mDA neurons in SNc versus VTA differ in the timing of peak neurogenesis (Fig. 1*A*; Bayer et al., 1995; Bye et al., 2012).

To examine whether progressive competence restriction may contribute to the generation of diverse mDA subpopulations, we used an inducible genetic fate-mapping approach that relies on the expression of *Cxcr4* (CXC motif chemokine receptor 4). Using *Cxcr4^CreER^* mice (Werner et al., 2020) in combination with classical reporter alleles or intersectional reporter strategies allows us to label mDA progenitors and precursors (and all their descendants) at different embryonic time points. The analysis of the distribution of fate-mapped neurons in prenatal and postnatal brains shows that the activation of Cre-mediated recombination by the administration of tamoxifen (TM) at different time points (between E11.5 and E15.5) results in progressively less contribution of fate-mapped cells to mDA neurons. Importantly, we demonstrate that the anatomic distribution and consequently the projection targets of mDA neurons derived from the *Cxcr4* lineage get more restricted at later time points of progenitor/precursor labeling. These data indicate that mDA progenitors can initially give rise to all types of mDA neurons but are more restricted in their fate at later stages of development, which is consistent with a progressive competence restriction model.

## Materials and Methods

### Animals

All mouse experiments were carried out with strict observance of protocols and guidelines approved by the University of Bonn Animal Care and Use Committee, Federal Government of Germany and European Union legislation. The protocols were approved by the Landesamt für Natur, Umwelt und Verbraucherschutz Nordrhein-Westfalen (Permit Number: 84-02.02.2016.A238). The mice were housed under controlled light (12 h light/dark cycle at an ambient temperature of 22°C). Water and mice chow were available *ad libitum*.

*Cxcr4^CreER(T2)-IRES-eGFP^* (*Cxcr4^CreER^*) mice were provided by Ralf Stumm (Werner et al., 2020). *Rosa26^Ai9^* (stock #007909; Madisen et al., 2010), Dat*^tTA^* (stock #027178; Chen et al., 2015), and Ai82D (*lgs7^TITLGFP^*; stock #023532; Madisen et al., 2015) mice were purchased from The Jackson Laboratory. For inducible fate-mapping studies, *Cxcr4^CreER/+^*; *Rosa26^Ai9/Ai9^* males were bred with CD1 wild-type females (Charles River) to generate *Cxcr4^CreER/+^*; *Rosa26^Ai9/+^* progeny. For intersectional fate mapping, *Cxcr4^CreER/+^*; Dat*^tTA/+^*; *lgs7^TITLGFP/TITLGFP^* males were bred with CD1 wild-type females (Charles River) to generate *Cxcr4^CreER/+^*; Dat*^tTA/+^*; *lgs7^TITLGFP^* progeny. Mice of either sex were used for experiments.

### Tamoxifen administration

TM (catalog #T-5648, Sigma-Aldrich) was dissolved in corn oil (catalog #C8264, Sigma-Aldrich) at the final concentration of 20 mg/ml. Day of vaginal plug was designated as E0.5. To induce Cre recombinase, TM (75 mg/kg body weight) was administered by oral gavage with feeding needles to a pregnant female at 12:00 P.M. of E11.5, E12.5, E13.5, E14.5, or E15.5.

### Tissue processing

#### Embryonic brains

Pregnant dams were killed by cervical dislocation, and pups were rapidly removed by uterine horns, placed in ice-cold PBS, and decapitated. Embryonic brains were fixed in 4% paraformaldehyde (PFA) in PBS at 4°C overnight, cryoprotected in sucrose (15% sucrose in PBS followed by 30% sucrose in PBS), and embedded in Tissue Tek. Embryonic brains were sectioned at 14 μm, mounted on a coated glass slide, and stored at −20°C.

#### Adult brains (postnatal day 30)

Mice were anesthetized with an intraperitoneal injection of Ketanest/Rompun and subsequently perfused transcardially with PBS, followed by 4% PFA. Dissected brains were postfixed in PFA overnight at 4°C, cryoprotected in sucrose (15% sucrose in PBS followed by 30% sucrose in PBS) and mounted in Tissue Tek. Brains were sectioned coronally at 40 μm, and the free-floating sections were stored in antifreeze solution (30% glycerol and 30% ethylene glycol in phosphate buffer) at −20°C.

### Immunofluorescence

Sections of embryonic brains were thawed for 2 h at room temperature (RT), rinsed in PBS at RT, postfixed in 4% PFA for 5 min, and again rinsed in PBS followed by PBS + 0.5% Triton X-100 (PBT). Then they were incubated in PBT plus 10% normal donkey serum (NDS; Jackson ImmunoResearch) at RT for 2 h, followed by primary antibodies (Table 1) in PBT + 3% NDS at 4°C overnight. After washing in PBT, sections were incubated with secondary antibodies (Table 1) in PBT + 3% NDS for 2 h at RT. Hoechst 33258 (Abcam) was used to counterstain nuclei. Sections of adult brains were rinsed in PBS at RT and then incubated in PBS + 0.5% Triton X-100 (PBT) plus 10% NDS (Jackson ImmunoResearch) at RT for 2 h. Sections were incubated with primary antibodies in PBT + 3% NDS at 4°C overnight. After washing in PBT, sections were incubated with secondary antibodies in PBT + 3% NDS for 2 h at RT. Hoechst 33258 (Abcam) was used to counterstain nuclei.

**Table 1:** Antibodies used in this study

Antibodies	Source	Identifier	Dilution
Rabbit anti-RFP	Rockland	RRID:AB_2209751	1:1000 or 1:5000
Mouse anti-TH	Merck	RRID:AB_2201528	1:400 or 1:500
Rabbit anti-TH	Merck	RRID:AB_390204	1:500–1:1000
Mouse anti-glutamine synthetase	Merck	RRID:AB_2110656	1:500
Rat anti-GFP	Nacalai Tesque	RRID:AB_10013361	1:2000
Donkey anti mouse-Alexa Fluor 488	Thermo Fisher Scientific	RRID:AB_141607	1:500
Donkey anti mouse-Alexa Fluor 647	Thermo Fisher Scientific	RRID:AB_162542	1:500
Donkey anti rabbit-Alexa Fluor 488	Thermo Fisher Scientific	RRID:AB_2535792	1:500
Donkey anti rabbit-Alexa Fluor 546	Thermo Fisher Scientific	RRID:AB_2534016	1:500
Donkey anti rabbit-Alexa Fluor 647	Thermo Fisher Scientific	RRID:AB_2536183	1:500
Donkey anti rat-Alexa Fluor 488	Thermo Fisher Scientific	RRID:AB_2535794	1:500
Donkey anti-rabbit Biotin-SP	Jackson ImmunoResearch	RRID:AB_2340593	1:200
Streptavidin Cy3	Jackson ImmunoResearch		1:200

### Imaging

Images of fluorescently stained sections were acquired with an inverted microscope (model AxioObserver Z1, Carl Zeiss) equipped with structured illumination (ApoTome) and a camera (model AxioCam MRm, Carl Zeiss). At 10× magnification (EC PlnN 10×/0.3; Carl Zeiss), tile images were acquired with conventional epifluorescence. At 20× magnification (EC PlnN 20×/0.5; Carl Zeiss), 40× magnification (Pln Apo 40×/1.3 Oil; Carl Zeiss), and 63× magnification (Pln Apo 63×/1.4 Oil; Carl Zeiss), structured illumination was used to acquire tile images and *z*-stacks. Some of the images taken with the 20× objective and all the images taken with the 40× and 63× objectives are maximum-intensity projections of *z*-stacks. Tile images were stitched with Zen Blue software (version 2012; Carl Zeiss). The images are presented either as maximum intensity projection of the *z*-stacks or as single optic layers.

### Quantification of recombined mDA neurons in adult brains

Quantification of tdTomato (tdT)-expressing recombined mDA neurons (in *Cxcr4^CreER^ Rosa26^Ai9^* animals) were quantified at one rostrocaudal level (from bregma, −3.52 mm). mDA-containing areas were outlined for each level (medial or lateral SNc; ventral or dorsal VTA) and TH-positive cells and TH-, tdT-double-positive cells were counted. For each outlined area, the number of fate-mapped mDA neurons (TH-, tdT-double positive) was normalized for the total number of TH-positive cells.

Recombined mDA neurons in the intersectional labeling approach were quantified on coronal sections at four rostrocaudal levels for four animals at (level 1: bregma, −2.70 mm; level 2, −3.16 mm; level 3, −3.52 mm; level 4, −3.80 mm). mDA-containing areas were outlined for each level (medial, lateral, or caudal SNc; rostral, ventral, or dorsal VTA) and TH-positive cells and TH-, GFP-double-positive cells were counted. For each outlined area, the number of fate-mapped mDA neurons (TH-, GFP-double positive) was normalized for the total number of TH-positive cells or for the number of GFP-positive cells.

### Statistical analysis

Statistical analyses of cell numbers were performed with GraphPad Prism (9.0) software using one-way ANOVA followed by Šídák's multiple-comparison test. Sample size estimation was not performed. No data or subjects were excluded from the analysis. Statistical details are described in the figure legends or in the results. *p* values of <0.05 were considered statistically significant. Data are reported as mean values ± SEM.

## Results

### Inducible fate mapping of *Cxcr4*-expressing progenitors and mDA precursors

**Figure 2.**
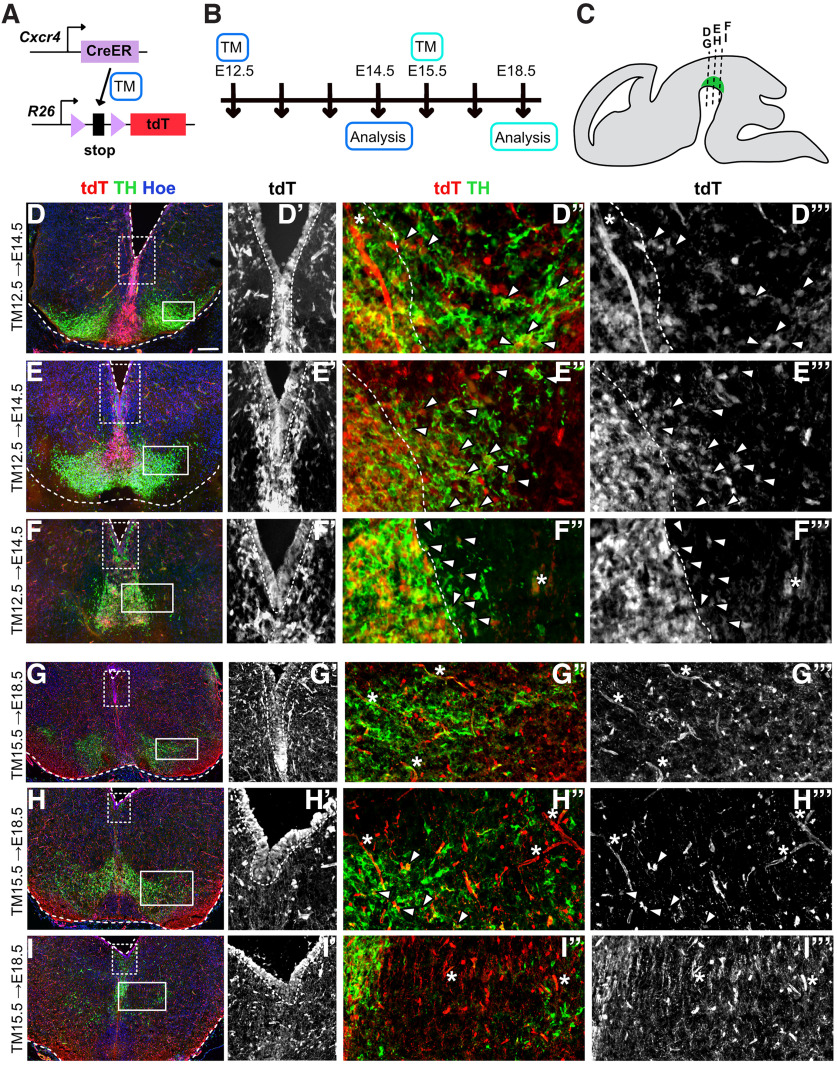
*Cxcr4*-inducible fate-mapping strategy results in the labeling of mDA progenitors and precursors. ***A***, Fate-mapping strategy to label *Cxcr4*-expressing cells and their descendants with tdT. *Cxcr4^CreER^* mice were used in combination with the reporter mouse line *Ai9*. CreER is activated by TM administration. ***B***, Experimental timeline. ***C***, Sagittal view of the embryonic mouse brain. mDA neurons are indicated in green. Dotted lines indicate levels of sections in ***D-I′′′***. ***D, E, F, G, H, I***, Immunostaining for tdT (red) and TH (green) on E14.5 (***D***, ***E***, ***F***) or E18.5 (***G***, ***H***, ***I***) coronal midbrain sections. Counterstain: Hoechst (Hoe). ***D*′**, ***E*′**, ***F*′**, ***G*′**, ***H*′**, ***I*′**, Ventricular zone is outlined. Area indicated by the box (dashed line) in ***D***, ***E***, ***F***, ***G***, ***H***, ***I***. ***D*′′, *E*′′, *F*′′, *G*′′**, ***H*′′, *I*′′,** and ***D*′′′**, ***E*′′′**, ***F*′′′**, ***G*′′′**, ***H*′′′**, and ***I*′′′**, Area indicated by the box (solid line) in ***D***, ***E***, ***F***, ***G***, ***H***, and ***I***. Asterisks indicate blood vessels. ***D*′′**, ***E*′′**, ***F*′′** and ***D*′′′**, ***E*′′′**, ***F*′′′**, With TM12.5, medial mDA regions (area left of the dashed line) contain many tdT and TH double-positive cells. Arrowheads indicate tdT and TH double-positive cells in lateral mDA-containing areas (to the right of the dashed line). ***G*′′**, ***H*′′**, and ***I*′′**, and ***G*′′′**, ***H*′′′**, and ***I*′′′**, With TM15.5, few tdT and TH double-positive cells are present and only at intermediate levels (arrowheads). Scale bar: ***D***, ***E***, ***F***, ***G***, ***H***, ***I***, 100 μm. In addition, the contribution of the *Cxcr4* lineage to the midbrain ventricular zone was analyzed at E18.5 for the TM administration time points E11.5–E14.5 (Extended Data Fig. 2-1).

CXCR4 is widely expressed in the developing nervous system (Tissir et al., 2004). In the midbrain, the *Cxcr4* transcript is weakly, but broadly, expressed in ventral progenitors from E10.5 to at least E13.5. In addition, both the protein and the transcript are transiently expressed in newly differentiated, radially migrating mDA precursors just after they exit the ventricular zone. Slightly more mature mDA neurons have downregulated *Cxcr4*, while expression of the CXCR4 protein appears to persist for a while (Fig. 1*B*; Yang et al., 2013; Bodea et al., 2014). Based on the presence of *Cxcr4* in progenitors and the transient expression of *Cxcr4* in mDA precursors, labeling cells of the *Cxcr4* lineage at different developmental stages should make it possible to examine the competence of mDA progenitors/precursors at different developmental time points (Fig. 1*C*). This requires labeling *Cxcr4*-expressing progenitors/precursors in such a way that the labeling is retained after *Cxcr4* expression ceases (Fig. 1). To achieve such specific labeling, we combined a *Cxcr4^CreER^* mouse line with the reporter line *Ai9* that expresses tdT on recombination (genotype, *Cxcr4^CreER/+,^ R26^Ai9/+^*; Madisen et al., 2010; Werner et al., 2020). Administration of tamoxifen at distinct developmental time points (e.g., at E12.5, referred to as TM12.5) results in Cre activation, and recombination occurs in Cre-expressing (*Cxcr4*-expressing) cells approximately within a day after TM administration (Legué and Joyner, 2010). Once the *Ai9* allele is recombined, tdT expression is permanently turned on (Fig. 2*A*).

10.1523/ENEURO.0052-22.2022.f2-1 Figure 2-1 *Cxcr4*-inducible fate-mapping strategy results in labeling of cells in the midbrain ventricular zone between E11.5 and E14.5. ***A***, Experimental timeline. ***B***, Schematic of E18.5 ventral midbrain. The ventricular zone (VZ) and mDA neurons (green) are indicated. The dashed outline indicates the areas shown in ***C***, ***D***, ***E***, and ***F***. ***C***, ***D***, ***E***, ***F***, Immunostaining for tdT (red) and TH (green) on E18.5 coronal midbrain sections. Counterstain: Hoechst (Hoe). ***C*′′′, *D*′′′**, ***E*′′′**, ***F*′′′**, Areas indicated by the box in ***D*′′**, ***E*′′**, and ***F*′′**. Note that with TM11.5, almost all cells outside of the VZ express tdT; thus, the labeling of VZ cells is not as obvious as at subsequent time points of TM administration. Scale bars: ***C***, ***C*′′**, ***E***, ***E*′′**, ***F***, ***F*′′**, ***G***, ***G*′′**, 100 μm; ***C*′′′**, ***D*′′′**, ***E*′′′**, ***F*′′′**, 50 μm. Download Figure 2-1, TIF file.

To confirm that this approach does indeed result in the labeling of ventral midbrain progenitors and precursors, TM was administered at E12.5 and E15.5 and the fate-mapped (tdT-expressing) cells were analyzed shortly after onset of Cre-mediated recombination at E14.5 or E18.5, respectively (Fig. 2*B*). Immunostaining for TH and tdT at three rostrocaudal levels (Fig. 2*C*) showed extensive tdT expression in progenitors in the ventricular zone of the midbrain at both stages analyzed (Fig. 2*D*,*D*′,*E*,*E*′,*F*,*F*′,*G*,*G*′,*H*,*H*′,*I*,*I*′, Extended Data Fig. 2-1). With TM12.5, tdT was also broadly expressed in the area just below the ventralmost progenitor zone that corresponds to the region that contains TH-negative mDA precursors in rostral and intermediate levels of the ventral midbrain (Fig. 2*D*,*E*). In addition, TH-positive mDA neurons expressed tdT. Overlap of TH and tdT was particularly widespread in mDA neurons located close to the TH-negative mDA precursors, suggesting that the upregulation of TH expression was likely a relatively recent event in the tdT-positive neurons (Fig. 2*D–E*′′). Interestingly, at caudal levels, the TH-negative transition zone between the ventricular zone and TH-expressing neurons was essentially missing, and there was a broad overlap between tdT and TH (Fig. 2*F–F*′′′). Analysis of cells labeled with TM15.5 showed that scattered tdT-positive cells were located throughout the midbrain at E18.5 (Fig. 2*G–I′′′*). Although mDA neurogenesis is complete at E15.5, the labeling of *Cxcr4*-expressing cells resulted in a few TH and tdT double-positive neurons at intermediate levels, suggesting that these are derived from *Cxcr4*-expressing mDA precursors. Finally, we confirmed that mDA progenitors are also labeled at other time points of TM administration by administering TM between E11.5 and E14.5 and analyzing the fate-mapped (tdT-expressing) cells at E18.5 (Extended Data Fig. 2-1). We found that at all TM administration time points, cells in the ventricular zone were extensively labeled (Fig. 2, Extended Data Fig. 2-1). Labeling was not restricted to the ventral midbrain progenitors but was found throughout the ventricular zone, indicating that *Cxcr4* is broadly expressed in midbrain progenitors at the analyzed induction time points.

In summary, these data indicate that the *Cxcr4*-based lineage analysis allows the labeling of midbrain progenitors and mDA precursors, consistent with the expression pattern of *Cxcr4* (Yang et al., 2013; Bodea et al., 2014). The expression of tdT in TH-expressing mDA neurons 2 or 3 d after TM administration (TM12.5; analysis at E14.5 or TM15.5; analysis at E18.5) likely indicates that these cells have switched on TH expression between the onset of tdT expression in mDA precursors and the time point of analysis. Alternatively, some recombination could also occur in mDA neurons that have recently turned on TH expression.

### Contribution of *Cxcr4*-expressing progenitors and precursors to mDA neurons becomes more restricted over time

**Figure 3.**
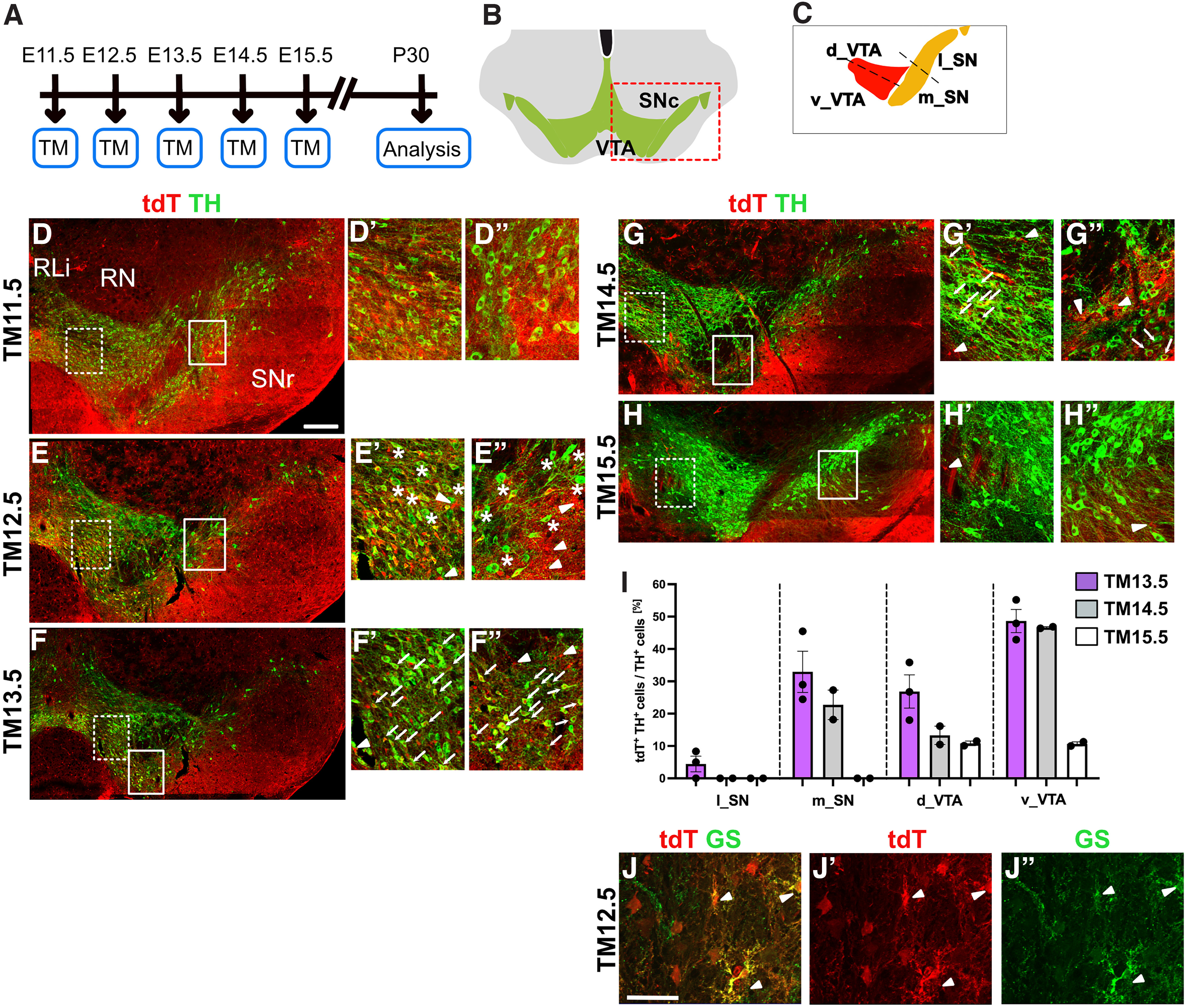
The *Cxcr4* lineage contributes to mDA neurons between E11.5 and E15.5. ***A***, Experimental timeline. ***B***, Coronal view of the adult ventral midbrain. mDA neurons are indicated in green. Red box indicates area shown in ***D***, ***E***, ***F***, ***G***, and ***H***. ***C***, Schematic indicating the medial SNc (m_SN), lateral SNc (l_SN), dorsal VTA (d_VTA), and ventral VTA (v_VTA). Fate-mapped cells in these areas are quantified in ***I***. ***D***, ***E***, ***F***, ***G***, ***H***, Immunostaining for tdT (red) and TH (green) on P30 coronal midbrain sections. Rli, Rostral linear nucleus; RN, red nucleus. ***D*′**, ***E*′**, ***F*′**, ***G*′**, ***H*′**, Area in the VTA indicated by the box (dashed line) in ***D***, ***E***, ***F***, ***G***, and ***H***. ***D*′′**, ***E*′′**, ***F*′′**, ***G*′′**, ***H*′′**, Area in the SNc indicated by the box (solid line) in ***D***, ***E***, ***F***, ***G***, and ***H***. ***E*′–*H*′′**, Arrowheads indicate tdT-expressing cells with glial morphology. ***D–D*′′**, With TM11.5, most cells in the ventral midbrain appear to be positive for tdT, including almost all mDA neurons. ***E–E*′′**, With TM12.5, some mDA neurons do not coexpress tdT (indicated by asterisks). ***F–G*′′**, With TM13.5 and TM14.5, many tdT-positive mDA neurons (indicated by arrows) are present in the ventral part of the VTA. In the SNc, double-labeled mDA neurons are almost completely restricted to the medial SNc. ***H–H*′′**, With TM15.5, only very few tdT-expressing mDA neurons are present and none are found in the examples in ***H*′** and ***H*′′**. ***I***, Quantitative analysis of tdT-expressing cells in the P30 midbrain. The percentage of tdT-expressing TH-positive mDA neurons was determined in m_SN, l_SN, d_VTA, and v_VTA as shown in the schematic in ***C***. With TM13.5, TM14.5, or TM15.5, the *Cxcr4* lineage does essentially not contribute to mDA neurons in the l_SN. With TM15.5, the few labeled mDA neurons are restricted to the d_VTA and v_VTA. ***J–J*′′**, Immunostaining for tdT (red) and glutamine synthetase (GS; green), an astrocyte marker on P30 coronal midbrain sections. TM was administered at E12.5. Scale bars: ***D***, ***E***, ***F***, ***G***, ***H***, 200 μm; ***J***, 50 μm. Analysis of the contribution of the *Cxcr4* lineage to mDA neurons and other midbrain areas at E18.5 (Extended Data Figs. 3-1, 3-2).

To gain a comprehensive overview of the extent of labeling at different developmental time points, the distribution of fate-mapped (tdT-expressing) cells was analyzed in E18.5 and postnatal day 30 (P30) brains from mice that received TM between E11.5 and E15.5. To examine the contribution of the fate-mapped cells to mDA neurons, we performed immunostaining for tdT and TH. Both at E18.5 and in P30 brains, it was obvious that the contribution of the *Cxcr4* lineage to the ventral midbrain was very broad at the earliest induction time point (TM11.5) and became progressively more restricted (Fig. 3, Extended Data Fig. 3-1). With TM11.5, the *Cxcr4* lineage did not only contribute broadly to mDA neurons, but also to the red nucleus, the SN pars reticulata (SNr), the TH-negative parts of the rostral and caudal linear nucleus, and other regions of the ventral and dorsal midbrain (Fig. 3, Extended Data Figs. 3-1, 3-2). Contribution to these areas became sparser over subsequent time points of TM administration.

10.1523/ENEURO.0052-22.2022.f3-1 Figure 3-1 The *Cxcr4* lineage contributes to mDA neurons between E11.5 and E15.5. ***A***, Experimental timeline. ***B***, Coronal view of the E18.5 brain. mDA neurons are indicated in green. The dashed outline indicates the area shown in ***C***, ***D***, ***E***, ***F***, and ***G***. ***C***, ***D***, ***E***, ***F***, ***G***, Immunostaining for tdT (red) and TH (green) on E18.5 coronal midbrain sections. ***C*′–*G*′′**, Area in the VTA indicated by the box (dashed line) in ***D***, ***E***, ***F***, ***G***, and ***H***. ***C*′′′–*G*′′′′**, Area in the SNc indicated by the box (solid line) in ***D***, ***E***, and ***F***. ***C*–*C*′′′′**, With TM11.5, almost all mDA neurons appear to be labeled with tdT. In addition, most cells in the ventral midbrain appear to be positive for tdT. ***D–D*′′′′**, With TM12.5, few mDA neurons do not coexpress tdT (indicated by asterisks). ***E*–*F*′′′′**, With TM13.5 and TM14.5, many tdT-negative mDA neurons (indicated by asterisks) are detected in the dorsal part of the VTA. In the SNc, only a few double-labeled mDA neurons (indicated by arrowheads) are present. ***G*–*G*′′′′**, With TM15.5, only a few tdT-expressing mDA neurons (indicated by arrowheads) are present in the VTA, and double-labeled cells are essentially absent from the SNc. Scale bars: ***C***, ***D***, ***E***, ***F***, ***G***, 100 μm. Download Figure 3-1, TIF file.

10.1523/ENEURO.0052-22.2022.f3-2 Figure 3-2 The *Cxcr4* lineage contributes cells to the entire midbrain between E11.5 and E15.5. Analysis at E18.5. ***A***, Experimental timeline. ***B***, ***C***, ***D***, ***E***, ***F***, Immunostaining for tdT (red) and TH (green) on E18.5 coronal midbrain sections. Rli, Rostral linear nucleus; RN, red nucleus. ***B*′**, ***C*′**, ***D*′**, ***E*′**, ***F*′**, Area in the dorsal midbrain indicated by the box (dashed line) in ***C***, ***D***, ***E***, ***F***, and ***G***. ***B*′′**, ***C*′′**, ***D*′′**, ***E*′′**, ***F*′′**, Area including the RLi in the ventral midbrain indicated by the box (solid line) in ***B***, ***C***, ***D***, ***E***, and ***F***. ***B*–*B*′′**, With TM11.5, almost all cells in the selected areas appear to express tdT. ***B*–*F*′′**, The number of tdT-expressing cells decreases progressively from TM11.5 to TM15.5. Scale bar, 200 μm. Download Figure 3-2, TIF file.

Given the extensive labeling of cells in the ventral midbrain at TM11.5 and TM12.5 (Fig. 3*D–E′′*), it was not possible to assess the contribution of the *Cxcr4* lineage to different anatomic subsets of mDA neurons in a quantitative manner at these two induction time points. Qualitative and quantitative assessment of cell distribution at subsequent stages of TM induction showed that with TM13.5, tdT-positive mDA neurons were found primarily in the VTA and in the medial SNc, with little contribution of the fate-mapped cells to mDA neurons in the lateral SNc. With TM14.5, the highest percentage of tdT-positive mDA neurons was found in the ventral VTA, while by TM15.5 only very few VTA–mDA neurons were tdT positive (Fig. 3*C*,*F–I*, Extended Data Fig. 3-1). At all stages analyzed, the *Cxcr4* lineage gave rise to neurons and cells with glial morphology (Fig. 3*D–H*). Staining for glutamine synthetase, a marker for astrocytes, and tdT confirmed that a subset of cells with glial morphology were astrocytes (Fig. 3*J*).

### Specific labeling of mDA neurons derived from the *Cxcr4* lineage

**Figure 4.**
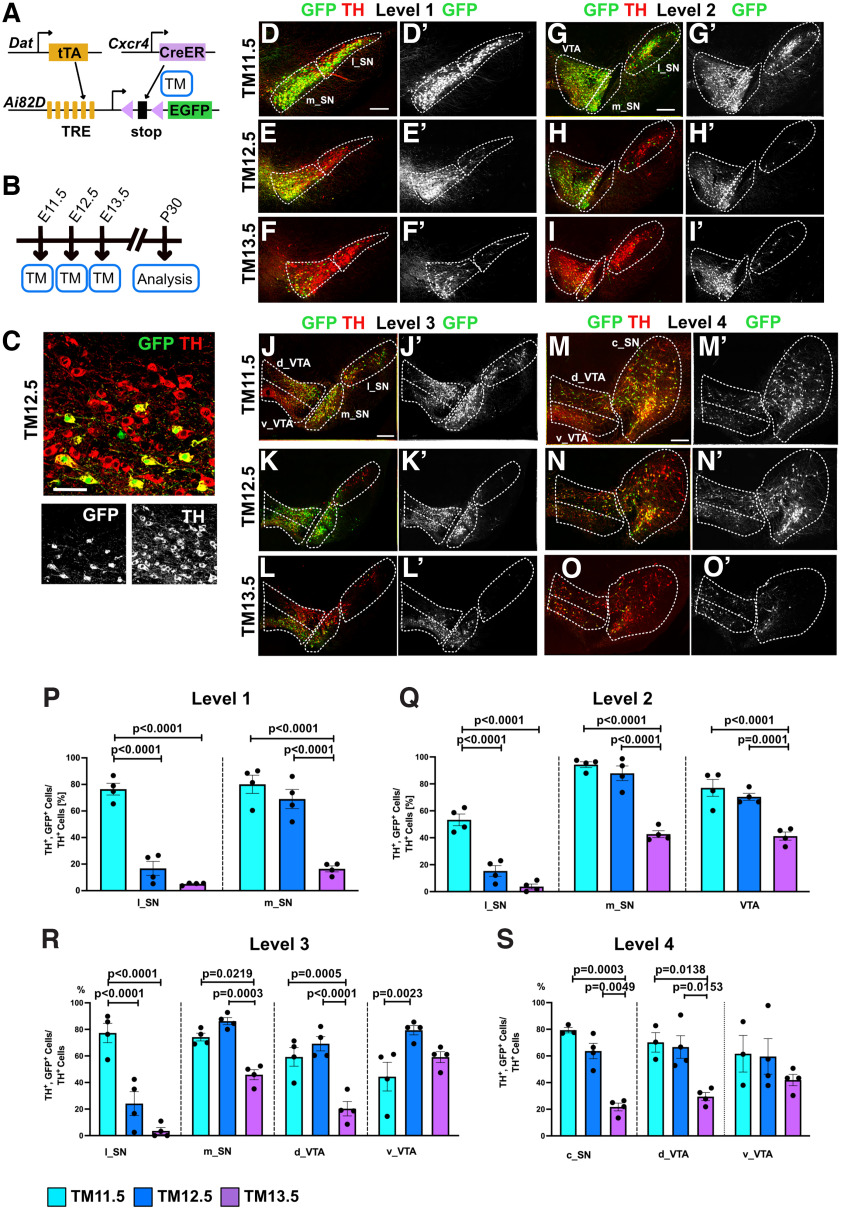
The *Cxcr4* lineage contributes to progressively more restricted populations of mDA neurons over the course of development. ***A***, Intersectional strategy to label *Cxcr4*-expressing mDA neurons with enhanced GFP (EGFP). tTA, Tetracycline controlled transactivator; TRE, tetracyclin response element. ***B***, Experimental timeline. ***C–O***, Immunostaining for TH (red) and GFP (green). ***D***′-***O***′, Immunostaining for GFP. ***C***, Higher magnification of an area in the VTA shows that almost all GFP-expressing cells coexpress TH (TM12.5). ***D–O′***, Coronal sections from 4 different rostrocaudal levels. Medial SNc (m_SN), lateral SNc (l_SN), caudal SNc (c_SN), dorsal VTA (d_VTA), and ventral VTA (v_VTA). ***P–S***, Contribution of fate-mapped cells to the levels and regions shown in ***D–O′***. Number of cells labeled with the intersectional approach is expressed as a percentage of the number of mDA neurons in each indicated region. Scale bars: ***C***, 50 μm; ***D–O***′, 200 μm. Higher-magnification images of selected areas in the SNc and VTA are shown in Extended Data Figure 4-1.

To label specifically mDA neurons of the *Cxcr4* lineage, we used an intersectional labeling approach. To this end, the *Cxcr4^CreER^* mouse line was used in conjunction with a *Dat^tTA^* mouse line [tetracycline transactivator expressed under control of the *Slc6a3* (*Dat*) locus] and the *Ai82* intersectional reporter mouse (Chen et al., 2015; Madisen et al., 2015; Poulin et al., 2018). Since the expression of *Dat* (dopamine transporter) is restricted to mDA neurons, tTA expression should be mDA neuron specific. In the *Ai82* mouse line, an enhanced green fluorescent protein (GFP) is expressed only in cells in which Cre-mediated recombination has occurred and that also express tTA (Fig. 4*A*). Indeed, immunostaining for GFP and TH showed that the large majority of GFP-expressing cells were also positive for the mDA marker TH when using this intersectional labeling approach (Fig. 4*C*), consistent with previous reports (Poulin et al., 2018).

10.1523/ENEURO.0052-22.2022.f4-1 Figure 4-1 The *Cxcr4* lineage contributes to progressively more restricted populations of mDA neurons over the course of development. ***A–L***, Immunostaining for tdT (red) and TH (green) on P30 coronal midbrain sections: medial SNc (m_SN), lateral SNc (l_SN), dorsal VTA (d_VTA), ventral VTA (v_VTA), and caudal SNc (c_SN). Note that the overview images (***A–L***, left panels) are the images that are also shown in Figure 4. Boxes indicate the higher-magnification areas shown to the right. Scale bar, 200 μm. Download Figure 4-1, TIF file.

Since our analysis with the *Ai9* reporter allele showed that the *Cxcr4* lineage gives rise to mDA neurons in the SNc and VTA primarily between TM11.5 and TM13.5 (Fig. 3, Extended Data Fig. 3-1), we focused on these TM administration time points for the intersectional approach (Fig. 4*B*). Brains were analyzed at P30. To investigate the anatomic distribution of *Cxcr4* lineage-derived mDA neurons (GFP expressing), immunostaining for GFP and TH was performed on four coronal levels along the rostrocaudal axis of the adult midbrain (Fig. 4*D–O*; see also Materials and Methods). Quantification of the number of GFP-positive cells confirmed our previous observation obtained using the *Ai9* allele: the number of GFP-expressing mDA neurons was similar with TM11.5 and TM12.5 but was greatly reduced with TM13.5 (number of GFP-positive cells: at TM11.5, 812.75 ± 34.04; at TM12.5, 702.5 ± 25.69; at TM13.5, 280 ± 41.0).

Next, we assessed whether the contribution of the fate-mapped cells to different anatomic areas of the SNc and VTA changed with different labeling time points. The SNc was divided into medial and lateral SNc (level 1–3) and caudal SNc (level 4); and the VTA into rostral VTA (level 2) and dorsal and ventral VTA at levels 3 and 4 (Figs. 3*C*, 4*D–O*, Extended Data Fig. 4-1). The SN pars lateralis (SNpl) was not assessed separately but was included in the lateral SNc. To account for differences in the numbers of mDA neurons in these various areas, the number of TH- and GFP-double positive cells was normalized for the number of TH-positive cells for each area (e.g., for medial SNc at level 1). When comparing TM11.5 and TM12.5, there was no significant change in the contribution of fate-mapped cells to mDA neurons in the medial SNc, rostral VTA, dorsal VTA, or caudal SNc. However, we found a strong reduction in the contribution of labeled cells to the lateral SNc and a significant increase in the percentage of TH- and GFP-positive mDA neurons in the ventral VTA at level 3 (Fig. 4*D–S*, Extended Data Fig. 4-1). With TM13.5, the contribution of fate-mapped cells to mDA neurons in the medial SNc, rostral VTA, dorsal VTA, and caudal SNc was significantly reduced compared with the previous two time points of TM induction. However, there was no significant change in the percentage of fate-mapped cells contributing to mDA neurons in the ventral VTA. Only a few TH- and GFP-positive neurons were located in the lateral SNc (Fig. 4*D–S*, Extended Data Fig. 4-1). These data suggest that when labeled with TM11.5, the *Cxcr4* lineage gives rise to essentially all subtypes of mDA neurons in the SNc and VTA. With TM12.5, *Cxcr4*-expressing mDA progenitors/precursors still have the potential to develop into most mDA neurons except for the lateral SNc. With TM13.5, despite the reduced overall contribution of the *Cxcr4* lineage to mDA neurons, the percentage of TH- and GFP-expressing neurons in the ventral VTA remains constant, suggesting that at this stage the mDA progenitors/precursors fate is largely restricted to mDA neurons in the ventral VTA.

### Projection pattern of mDA neurons in the *Cxcr4* lineage

Since we observed a restricted anatomic distribution of fate-mapped mDA neurons with TM12.5 and TM13.5, we next examined whether these anatomically restricted populations show specific innervation patterns of the forebrain target regions of mDA neurons. Forebrain sections from six rostrocaudal levels were costained for TH to delineate the entire area innervated by mDA neurons and for GFP to visualize projections of fate-mapped mDA neurons. The analysis was focused on the striatum, and other mDA neuronal targets such as PFC, lateral septum, and amygdala were not assessed.

**Figure 5.**
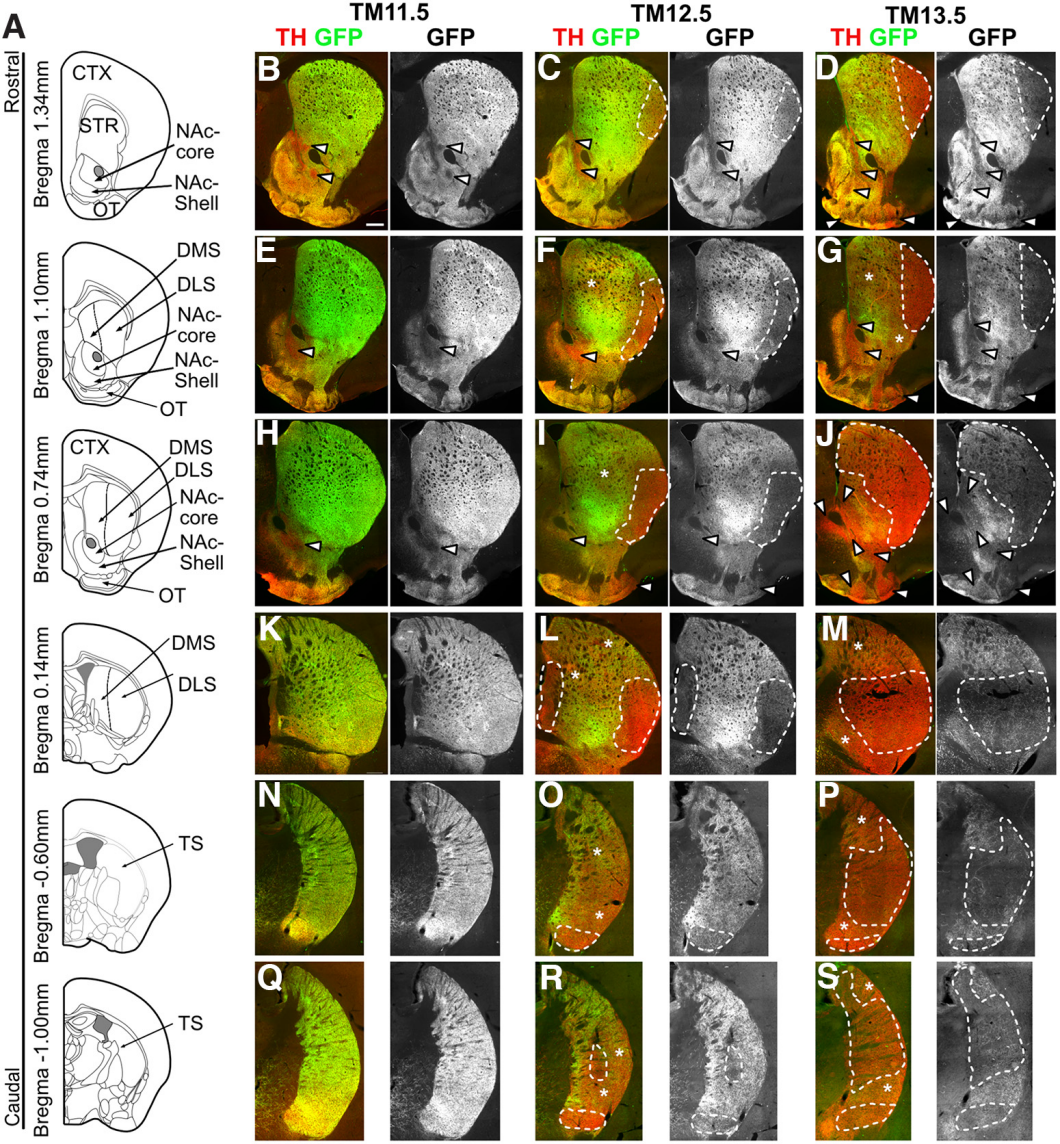
The progressively more restricted contribution of the *Cxcr4* lineage to mDA neurons is reflected in the projection pattern of the fate-mapped mDA neurons. mDA neurons were labeled using the intersectional approach described in Figure 4 with TM11.5, TM12.5 and TM13.5. ***A***, Levels shown in ***B–S***. ***B–S***, Immunostaining for TH and GFP. Arrowheads indicate areas with little or no innervation of GFP-positive fibers within the NAc or OT. Dashed lines indicate areas with little or no innervation of GFP-positive fibers, while asterisks indicate areas with intermediate density innervation of GFP-positive fibers in the DLS or DMS or TS. Scale bar, 500 μm. Higher-magnification images of selected projection target areas are shown in Extended Data Figure 5-1.

The projections of GFP-expressing mDA neurons labeled with TM11.5, appeared dense throughout the DMS, DLS, and tail of the striatum (TS). GFP-positive innervation of the NAc shell and olfactory tubercle (OT) appeared sparser than in the dorsal striatum and was partially absent from the NAc core (Fig. 5, Extended Data Fig. 5-1*A–D*′). With TM12.5, GFP-positive projections were less dense in the TS and almost absent in the most lateral part of the DLS (Fig. 5, Extended Data Fig. 5-1*E–H*′′). This is consistent with the very low contribution of fate-mapped cells to the lateral SNc and SNpl with TM12.5, since the DSL and TS are the two main projection targets of mDA neurons in the lateral SNc and SNpl, respectively (Lerner et al., 2015; Menegas et al., 2018). The GFP-positive innervation of the DMS, a target of mDA neurons in the medial SNc, and of the NAc and OT showed a similar pattern with TM12.5 and TM11.5 (Fig. 5), again consistent with the distribution of GFP-positive cells and previous studies (Beier et al., 2015; Lerner et al., 2015). With TM13.5, the GFP-positive innervation of the DLS was even more restricted than with TM12.5. Interestingly, while the DMS was still strongly positive for GFP at rostral levels, innervation was substantially reduced at intermediate and caudal levels, and innervation of the TS was very sparse. The density of GFP-expressing projections to the NAc and OT was comparable with TM12.5 and TM11.5 (Fig. 5, Extended Data Fig. 5-1*I–M*′′). Thus, the progressively more restricted competence of the *Cxcr4*-expressing mDA progenitors/precursors is also reflected in the more and more restricted innervation pattern of mDA neuronal targets in the striatum.

10.1523/ENEURO.0052-22.2022.f5-1 Figure 5-1 Innervation of projection targets by mDA neurons of the *Cxcr4*-lineage. (***A–K′′***) Immunostaining for tdT (red) and TH (green) on P30 coronal sections. Areas outlined by dashed lines indicate regions that are essentially devoid of GFP-positive axons (***B′,B′′, F′,F′′, K′,K′′***). Note that these areas are surrounded by regions with low density GFP-positive innervation. In these low density innervation areas, GFP- and GFP+ axons are intermingled (***C, C′***). In areas with the highest density of GFP-positive fibers, the large majority of fibers appear to be GFP-positive (***D, D′, F***). (***E,G***) Higher magnification of selected areas in the overview images shown in Figure 5 (E corresponds to Figure 5L, G to Figure 5R). Scale bar: 500 μm. Download Figure 5-1, TIF file.

## Discussion

**Figure 6.**
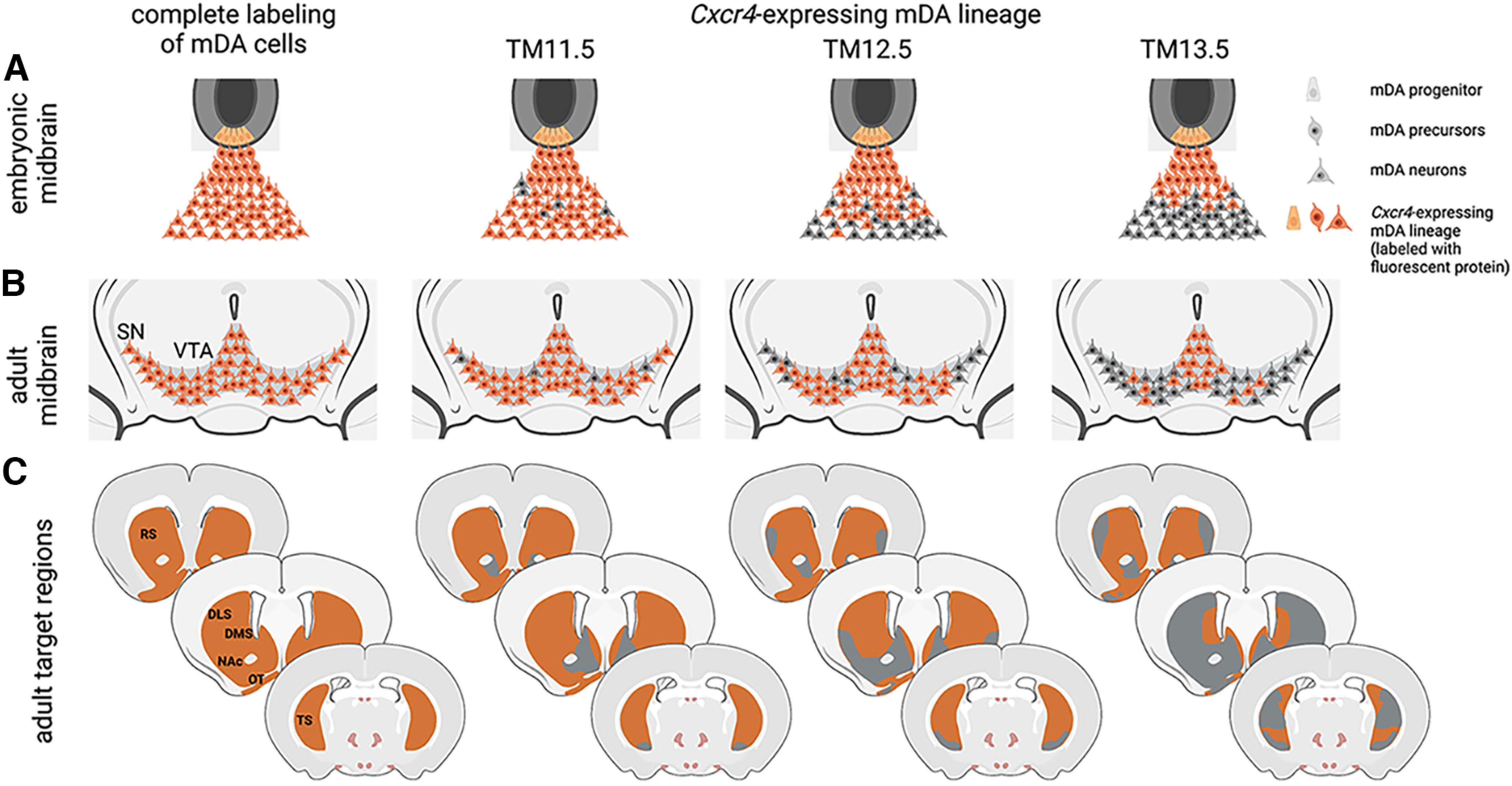
Summary of the results. ***A***, ***B***, The *Cxcr4*-expressing mDA lineage (***A***) contributes to progressively more restricted anatomic populations of mDA neurons (***B***) over the course of development. ***C***, The progressively more restricted contribution is also reflected in the projection pattern of the fate-mapped mDA neurons. Three rostrocaudal levels of the striatal region are shown. Gray regions, sparsely or not at all innervated by mDA neurons of the *Cxcr4* lineage; orange regions, densely innervated by mDA neurons of the *Cxcr4* lineage at the indicated time point of TM administration. This figure was created with https://biorender.com/.

mDA neurons are generated over several days of embryonic development (E10.0–E14.5 in the mouse), suggesting that the mDA neuronal population could be subdivided into cohorts based on the time window of their differentiation (Fig. 1*A*). Birth-dating studies indicate that the anatomic position of mDA neurons within the SNc and VTA correlates approximately with their time point of differentiation (Bayer et al., 1995; Bye et al., 2012), but how precisely these cohorts correlate with diverse anatomic populations of mDA neurons and subcircuits within the dopaminergic system has not been addressed. Here we use genetic inducible fate mapping to trace *Cxcr4*-expressing mDA progenitors and precursors at different developmental time points to assess the anatomic distribution and projection targets of their descendants. In contrast to birth dating, our fate-mapping strategy does not label cohorts with a specific birth date, but rather all descendants of the *Cxcr4*-expressing progenitors/precursors that were marked during the time window of Cre activity. Thus, if *Cxcr4* is expressed in all mDA progenitors, mDA progenitors that are fate mapped early—before or just at the onset of neurogenesis—should give rise to the entire population of mDA neurons. Progenitors that are labeled at consecutively later stages—after the onset of neurogenesis—are expected to give rise to a progressively smaller mDA neuronal population (Figs. 1*A*,*C*, 6*A*). Our data demonstrate that initially *Cxcr4*-expressing mDA progenitors and precursors have indeed the potential to give rise to a broad range of mDA neurons (TM11.5). At later stages of development, *Cxcr4*-expressing mDA progenitors and precursors give rise to a subset of mDA neurons. Importantly, this subset can be defined by its anatomic location (e.g., preferential contribution to the ventral VTA and medial SNc with TM13.5) and specific projection pattern (Fig. 6). Eventually, *Cxcr4*-expressing progenitors/precursors cease to contribute to mDA neurons (TM15.5). Together, these results point to a changing competence of mDA progenitors over time that leads to the generation of anatomically segregated mDA subtypes at different developmental time points. Such a progressive competence restriction is thought to underlie the generation of distinct projection neurons in the cerebral cortex (Llorca and Marín, 2021).

Since our labeling approach does not follow the fate of mDA progenitors at a single-cell resolution, our data do not resolve whether (1) the same progenitor gives sequentially rise to different types of mDA neurons (e.g., lateral SNc at E11.5, dorsal VTA and medial SNc at E12.5, ventral VTA at E13.5), (2) whether different types of progenitors give rise to different types of neurons during different time windows, or (3) whether the two mechanisms are combined. A clonal labeling method (e.g., MADM; Contreras et al., 2021) would be necessary to investigate this further. Model three would integrate the distinct competence of progenitors in the medial and lateral mDA progenitor domain described previously with changes in progenitor competence over time (Blaess et al., 2011; Hayes et al., 2011; Panman et al., 2014). While medial progenitors contribute to the SNc and dorsal VTA, lateral progenitors contribute primarily to the ventral VTA. Thus, neurogenesis may start later and also cease later in the lateral than in the medial domain, and the lateral progenitors may be the main source for late-born mDA neurons that settle in the ventral VTA.

Regardless of which model reflects the actual determinants of mDA progenitor cell competence, it will be interesting to investigate in the future whether the changes in mDA progenitor cell competence are cell intrinsic (e.g., is there a change in transcription factor expression over time) or extrinsic (i.e., are the progenitors exposed to varying environmental signals over time that influence their competence) and which molecular mechanisms play a role. CXCR4 itself may even be a mediator of an extrinsic mechanism. The CXCR4 ligand CXCL12 is expressed in the meninges of the ventral midbrain and has previously been implicated in modulating the migration of mDA neurons. Inactivation of CXCR4-mediated signaling in mice results in subtle changes in the migration pattern of mDA neurons (Yang et al., 2013; Bodea et al., 2014; Hillmer et al., 2015). However, whether neurogenesis or subtype specification of mDA neurons is altered in these mouse mutants has not been investigated, in part because homozygous mutant mice die prenatally. Examples for CXCR4-mediated signaling influencing neurogenesis come from the postnatal dentate gyrus, where CXCL12 signals from granule neurons prevent dispersion and support maturation of newborn granule neurons (Abe et al., 2018).

The intersectional approach used in our study allows a characterization of the fate-mapped mDA neurons that goes beyond the mere assessment of the anatomic location of the cell bodies as the dopaminergic subcircuits formed by the labeled cells can also be determined. With respect to SNc neurons, we show that SNc neurons derived from progenitors/precursors labeled with TM12.5 are localized almost exclusively in the medial SNc, and densely innervate the dorsal striatum, with the exception of the lateral part of the DLS, where projections from fate-mapped mDA neurons are scarce. Parts of the TS are only sparsely innervated by the labeled projections. The projections of the medial SNc neurons labeled with TM13.5 show not only this lateral–medial gradient in innervation of the dorsal striatum but also a rostral–caudal one: whereas the medial–rostral striatum shows dense innervation by the projections of the fate-mapped cells, the caudal aspects of the striatum and the TS are sparsely innervated (Fig. 6). Our observation that medial SNc neurons project to the DMS is consistent with the study of Lerner et al. (2015), which used retrograde viral tracing to show that DLS-projecting SNc–mDA neurons are located in the lateral SNc, and DMS-projecting ones in the medial SNc. In turn, the sparse innervation of the TS observed with TM13.5 is consistent with data showing that the TS is innervated by mDA neurons in the SNpl (Menegas et al., 2018; Poulin et al., 2018). However, our data also suggest that some of the dopaminergic projections in the TS arise from mDA neurons other than the ones in the SNpl, since innervation from mDA neurons fate mapped with TM12.5 is quite dense in some areas of the TS, although mDA neurons of the SNpl are not labeled. The graded innervation of the dorsal striatum along the rostral–caudal axis by different mDA subtypes in the medial SNc has not previously been described and adds new insight into the diversity of SNc–mDA neurons.

While the restricted contribution of late-forming mDA neurons to the medial SNc fits well with the topographic projection map of mDA neurons, it does not correlate with the “vulnerability map” of the SNc. SNc mDA neurons are divided into a ventral tier that is more susceptible to neurodegeneration and toxic insults, and a dorsal tier that is more resilient (Kordower et al., 2013). A recent study has shown that the ventral tier mDA neurons, which express the transcription factor SOX6 in the adult brain, are derived from a *Sox6*-expressing medial mDA progenitor domain (Luppi et al., 2021). These data could indicate that the spatial location and molecular profile of a progenitor determines the position of mDA neurons in the ventral or dorsal SNc (and potentially also their vulnerability), while the timing of neurogenesis determines their position in the medial or lateral SNc (and their connectivity to the DMS vs DLS).

With regard to the VTA, there is also anatomic, molecular, electrophysiological, and functional evidence that the ventral and dorsal VTA (also referred to as medial and lateral) are distinct (Lammel et al., 2008; Poulin et al., 2018; de Jong et al., 2019). According to these studies, the ventral (or medial) VTA population projects to the NAc ventral and medial shells. This is consistent with our data, which show that the ventral VTA population that is marked with TM13.5 projects densely to the NAc shell but not to the core. Whether this population projects to other previously described targets of the ventral VTA mDA neurons such as the prefrontal cortex and the basolateral amygdala will have to be explored in the future. Moreover, further studies will have to clarify whether mDA neurons generated at different developmental time points do not only differ in their anatomic position and projection targets but also in their molecular profiles and function.

Finally, we demonstrate that *Cxcr4* is broadly expressed in midbrain progenitors and that descendants of *Cxcr4*-expressing progenitors contribute neurons and glia cells to various midbrain nuclei and structures. Interestingly, it seems that Cxcr4 is only expressed in both progenitors and neuronal precursors in the mDA-generating region, while it is restricted to progenitors in other areas of the midbrain (Yang et al., 2013). Thus, inducible fate mapping of *Cxcr4*-expressing progenitors could be used in other areas of the midbrain to assess changing competencies of progenitor cells, possibly even with greater temporal precision than in mDA neurons.

## Synthesis

Reviewing Editor: Lynda Erskine, University of Aberdeen Decisions are customarily a result of the Reviewing Editor and the peer reviewers coming together and discussing their recommendations until a consensus is reached. When revisions are invited, a fact-based synthesis statement explaining their decision and outlining what is needed to prepare a revision will be listed below. The following reviewer(s) agreed to reveal their identity: Rajeshwar Awatramani, Patricia Jensen.

Thank you for submitting your work to eNeuro. As you will see below, both reviewers are agreed that your work corroborates and extends previous findings using elegant lineage analyses approaches. In general the reviewers found the paper to be well written and the data sound. They have, however, some questions and suggestions for improving the manuscript prior to publication.

Below is a list of points that we are all agreed should be addressed in a revised manuscript:

1. Fig 1 shows a nice cartoon of the proposed model, including Cxcr4 expression. However, it would be worth showing expression of Cxcr4 at a couple of different embryonic stages (e.g. E11.5, 13.5). Additionally, given that Cxcr4 expression extends beyond mDA progenitor domain, Figure 1B is misleading. Perhaps relabeling as Cxcr4 expressing mDA?

2. “To confirm that this approach does indeed result in labeling of ventral midbrain progenitors, TM was administered between E11.5 and E15.5 and the fate-mapped (tdT-expressing) cells were analyzed at E18.5 (Figure 2B).” Fig 2 is not compelling, and could be eliminated. It would be better to analyze embryos at earlier stages. Fig. 3 partially addresses this, but could be extended further (e.g. 11.5->13.5 examining progenitors and postmitotic neurons). In addition, some of the colocalizations in Fig. 3 are difficult to visualize.

3. In the Fig 6 atlas schematics, can the authors clarify how the DLS and DMS were demarcated.

4. The authors’ intersectional approach allows mapping of axons from the mDA subpopulations labeled by induction of cre expression on different developmental days. These results are potentially very important for understanding mDA neuron diversity. Unfortunately, the authors’ conclusions are difficult to evaluate based on the low magnification images in Figure 6. The authors should include some high mag images from the regions with high innervation by GFP+ axons, as well as some from areas that they indicate have little or no innervation. Within a region (high or low density), do the GFP+/TH+ and GFP-/TH+ axons tend to be intermingled, or do they show discrete localization?

5. It would be helpful to the reader to provide a summary schematic of the overall findings, noting which populations are labeled at each timepoint and where they project.

6. In the abstract and introduction, the authors mention that mDA neurogenesis occurs from E10.0-E14.5 in the mouse. They also state that in the inducible fate mapping approach, Cxcr4+ progenitors/precursors are labeled for approximately a day after tamoxifen injection. That being the case, why was tamoxifen injection performed on embryonic days 11.5-15.5, which would seem likely to miss the earliest differentiating mDA neurons?

7. Materials and Methods indicate Bregma at which neurons were quantified, while Figure 5 labels level 1-4. The authors should explicitly state which Bregma corresponds to which numbered level.

8. Why do Materials and Methods indicate that tdTomato-expressing mDA neurons were quantified in “medial, lateral, or caudal SN, rostral, ventral, or dorsal VTA” (page 6, paragraph 2), but Figure 4 shows quantification only in medial SN, lateral SN, ventral VTA, and dorsal VTA.” I suspect a cut-and-paste error, because exactly the same sentence is repeated in page 6, paragraph 3.

9. Page 8, paragraph 2: It is not clear what data are not shown. If the data are important, they should be shown in either a main or extended data figure.

General comments from the reviewers:

This paper uses lineage analysis of Cxcr4 expressing cells in the midbrain to demonstrate a change in competence of midbrain progenitors during embryonic development. In doing so, they corroborate and extend previous publications using standard birthdating techniques showing that the SN cohort is born earlier than the VTA cohort (e.g. Bayer, 1995; Bye, 2012). However, by utilizing intersectional genetic strategies, they are also able to unambiguously map projections of temporally defined cohorts, an important addition to the literature. They find that in general, DA projections restrict along the M-L and R-C axes of the striatum, in accordance with restriction in lineage-labeled cells. Overall, this is an interesting, well written paper, with a few minor flaws.

In this manuscript, the authors investigate whether midbrain dopaminergic (mDA) neuron diversity is generated by progressive competence restriction from Cxcr4-expressing progenitors/precursors. They utilize a simple inducible fate mapping approach with a tamoxifen-responsive Cxcr4Cre and Cre-responsive reporter, followed by an elegant inducible and intersectional approach to confirm and extend their initial results. By mapping the anatomical location of mDA neurons that differentiate between E11.5 and E15.5, the authors conclude that the mDA progenitors become increasingly restricted over time with respect to their potential cell fates. The authors data seem sound, and their study contributes to our understanding of the developmental origins of mDA neurons. I have only a few questions and suggestions for more detailed analysis.

## References

[B1] Abe P, Wüst HM, Arnold SJ, van de Pavert SA, Stumm R (2018) CXCL12‐mediated feedback from granule neurons regulates generation and positioning of new neurons in the dentate gyrus. Glia 66:1566–1576. 10.1002/glia.23324 29537098

[B2] Bayer SA, Wills KV, Triarhou LC, Ghetti B (1995) Time of neuron origin and gradients of neurogenesis in midbrain dopaminergic neurons in the mouse. Exp Brain Res 105:191–199. 10.1007/BF00240955 7498372

[B3] Beier KT, Steinberg EE, DeLoach KE, Xie S, Miyamichi K, Schwarz L, Gao XJ, Kremer EJ, Malenka RC, Luo L (2015) Circuit architecture of VTA dopamine neurons revealed by systematic input-output mapping. Cell 162:622–634. 10.1016/j.cell.2015.07.015 26232228 PMC4522312

[B4] Blaess S, Bodea GO, Kabanova A, Chanet S, Mugniery E, Derouiche A, Stephen D, Joyner AL (2011) Temporal-spatial changes in Sonic Hedgehog expression and signaling reveal different potentials of ventral mesencephalic progenitors to populate distinct ventral midbrain nuclei. Neural Dev 6:29. 10.1186/1749-8104-6-29 21689430 PMC3135491

[B5] Bodea GO, Spille J-H, Abe P, Andersson AS, Acker-Palmer A, Stumm R, Kubitscheck U, Blaess S (2014) Reelin and CXCL12 regulate distinct migratory behaviors during the development of the dopaminergic system. Development 141:661–673. 10.1242/dev.099937 24449842

[B6] Bye CR, Thompson LH, Parish CL (2012) Birth dating of midbrain dopamine neurons identifies A9 enriched tissue for transplantation into Parkinsonian mice. Exp Neurol 236:58–68. 10.1016/j.expneurol.2012.04.002 22524988

[B7] Chen L, Xie Z, Turkson S, Zhuang X (2015) A53T human α-synuclein overexpression in transgenic mice induces pervasive mitochondria macroautophagy defects preceding dopamine neuron degeneration. J Neurosci 35:890–905. 10.1523/JNEUROSCI.0089-14.2015 25609609 PMC4300331

[B8] Contreras X, Amberg N, Davaatseren A, Hansen AH, Sonntag J, Andersen L, Bernthaler T, Streicher C, Heger A, Johnson RL, Schwarz LA, Luo L, Rülicke T, Hippenmeyer S (2021) A genome-wide library of MADM mice for single-cell genetic mosaic analysis. Cell Rep 35:109274. 10.1016/j.celrep.2021.109274 34161767 PMC8317686

[B9] de Jong JW, Afjei SA, Dorocic IP, Peck JR, Liu C, Kim CK, Tian L, Deisseroth K, Lammel S (2019) A neural circuit mechanism for encoding aversive stimuli in the mesolimbic dopamine system. Neuron 101:133–151.e7. 10.1016/j.neuron.2018.11.005 30503173 PMC6317997

[B10] Dumas S, Wallén-Mackenzie Å (2019) Developmental co-expression of Vglut2 and Nurr1 in a mes-di-encephalic continuum preceeds dopamine and glutamate neuron specification. Front Cell Dev Biol 7:307. 31850343 10.3389/fcell.2019.00307 PMC6892754

[B11] Hayes L, Zhang Z, Albert P, Zervas M, Ahn S (2011) Timing of Sonic hedgehog and Gli1 expression segregates midbrain dopamine neurons. J Comp Neurol 519:3001–3018. 10.1002/cne.22711 21713771 PMC3154975

[B12] Hillmer RE, Boisvert JP, Cucciare MJ, Dwinell MB, Joksimovic M (2015) Generation and characterization of mice harboring a conditional CXCL12 allele. Int J Dev Biol 59:205–209. 10.1387/ijdb.140348mj 26505253

[B13] Kordower JH, Olanow CW, Dodiya HB, Chu Y, Beach TG, Adler CH, Halliday GM, Bartus RT (2013) Disease duration and the integrity of the nigrostriatal system in Parkinson’s disease. Brain 136:2419–2431. 10.1093/brain/awt192 23884810 PMC3722357

[B14] Lammel S, Hetzel A, Häckel O, Jones I, Liss B, Roeper J (2008) Unique properties of mesoprefrontal neurons within a dual mesocorticolimbic dopamine system. Neuron 57:760–773. 10.1016/j.neuron.2008.01.022 18341995

[B15] Legué E, Joyner AL (2010) Genetic fate mapping using site-specific recombinases. Methods Enzymol 477:153–181. 10.1016/S0076-6879(10)77010-5 20699142 PMC4684171

[B16] Lerner TN, Shilyansky C, Davidson TJ, Evans KE, Beier KT, Zalocusky KA, Crow AK, Malenka RC, Luo L, Tomer R, Deisseroth K (2015) Intact-brain analyses reveal distinct information carried by SNc dopamine subcircuits. Cell 162:635–647. 10.1016/j.cell.2015.07.014 26232229 PMC4790813

[B17] Lim L, Mi D, Llorca A, Marín O (2018) Development and functional diversification of cortical interneurons. Neuron 100:294–313. 10.1016/j.neuron.2018.10.009 30359598 PMC6290988

[B18] Llorca A, Marín O (2021) Orchestrated freedom: new insights into cortical neurogenesis. Curr Opin Neurobiol 66:48–56. 10.1016/j.conb.2020.09.004 33096393

[B19] Luppi MP, Azcorra M, Caronia-Brown G, Poulin J-F, Gaertner Z, Gatica S, Moreno-Ramos OA, Nouri N, Dubois M, Ma YC, Ramakrishnan C, Fenno L, Kim YS, Deisseroth K, Cicchetti F, Dombeck DA, Awatramani R (2021) Sox6 expression distinguishes dorsally and ventrally biased dopamine neurons in the substantia nigra with distinctive properties and embryonic origins. Cell Rep 37:109975. 10.1016/j.celrep.2021.109975 34758317 PMC8607753

[B20] Madisen L, Zwingman TA, Sunkin SM, Oh SW, Zariwala HA, Gu H, Ng LL, Palmiter RD, Hawrylycz MJ, Jones AR, Lein ES, Zeng H (2010) A robust and high-throughput Cre reporting and characterization system for the whole mouse brain. Nat Neurosci 13:133–140. 10.1038/nn.2467 20023653 PMC2840225

[B21] Madisen L, et al. (2015) Transgenic mice for intersectional targeting of neural sensors and effectors with high specificity and performance. Neuron 85:942–958. 10.1016/j.neuron.2015.02.022 25741722 PMC4365051

[B22] Manno GL, Gyllborg D, Codeluppi S, Nishimura K, Salto C, Zeisel A, Borm LE, Stott SRW, Toledo EM, Villaescusa JC, Lönnerberg P, Ryge J, Barker RA, Arenas E, Linnarsson S (2016) Molecular diversity of midbrain development in mouse, human, and stem cells. Cell 167:566–580.e19. 10.1016/j.cell.2016.09.027 27716510 PMC5055122

[B23] Menegas W, Akiti K, Amo R, Uchida N, Watabe-Uchida M (2018) Dopamine neurons projecting to the posterior striatum reinforce avoidance of threatening stimuli. Nat Neurosci 21:1421–1430. 10.1038/s41593-018-0222-1 30177795 PMC6160326

[B24] Morales M, Margolis EB (2017) Ventral tegmental area: cellular heterogeneity, connectivity and behaviour. Nat Rev Neurosci 18:73–85. 10.1038/nrn.2016.165 28053327

[B25] Panman L, Papathanou M, Laguna A, Oosterveen T, Volakakis N, Acampora D, Kurtsdotter I, Yoshitake T, Kehr J, Joodmardi E, Muhr J, Simeone A, Ericson J, Perlmann T (2014) Sox6 and Otx2 control the specification of substantia nigra and ventral tegmental area dopamine neurons. Cell Rep 8:1018–1025. 10.1016/j.celrep.2014.07.016 25127144

[B26] Poulin J-F, Caronia G, Hofer C, Cui Q, Helm B, Ramakrishnan C, Chan CS, Dombeck DA, Deisseroth K, Awatramani R (2018) Mapping projections of molecularly defined dopamine neuron subtypes using intersectional genetic approaches. Nat Neurosci 21:1260–1271. 10.1038/s41593-018-0203-4 30104732 PMC6342021

[B27] Poulin J-F, Gaertner Z, Moreno-Ramos OA, Awatramani R (2020) Classification of midbrain dopamine neurons using single-cell gene expression profiling approaches. Trends Neurosci 43:155–169. 10.1016/j.tins.2020.01.004 32101709 PMC7285906

[B28] Roeper J (2013) Dissecting the diversity of midbrain dopamine neurons. Trends Neurosci 36:336–342. 10.1016/j.tins.2013.03.003 23582338

[B29] Tissir F, Wang C-E, Goffinet AM (2004) Expression of the chemokine receptor Cxcr4 mRNA during mouse brain development. Brain Res Dev Brain Res 149:63–71. 10.1016/j.devbrainres.2004.01.002 15013630

[B30] Wallén Å, Zetterström RH, Solomin L, Arvidsson M, Olson L, Perlmann T (1999) Fate of mesencephalic AHD2-expressing dopamine progenitor cells in nurr1 mutant mice. Exp Cell Res 253:737–746. 10.1006/excr.1999.4691 10585298

[B31] Werner Y, Mass E, Kumar PA, Ulas T, Händler K, Horne A, Klee K, Lupp A, Schütz D, Saaber F, Redecker C, Schultze JL, Geissmann F, Stumm R (2020) Cxcr4 distinguishes HSC-derived monocytes from microglia and reveals monocyte immune responses to experimental stroke. Nat Neurosci 23:351–362. 10.1038/s41593-020-0585-y 32042176 PMC7523735

[B32] Yang S, Edman LC, Sánchez-Alcañiz JA, Fritz N, Bonilla S, Hecht J, Uhlén P, Pleasure SJ, Villaescusa JC, Marín O, Arenas E (2013) Cxcl12/Cxcr4 signaling controls the migration and process orientation of A9-A10 dopaminergic neurons. Development 140:4554–4564. 10.1242/dev.098145 24154522

