# Supplementary figures and images for: Lineage Analysis of Cxcr4-Expressing Cells in the Developing Midbrain Suggests That Progressive Competence Restriction in Dopaminergic Progenitor Cells Contributes to the Establishment of Dopaminergic Neuronal Diversity
Source: eNeuro. 2022 Aug 23;9(4):ENEURO.0052-22.2022. doi: 10.1523/ENEURO.0052-22.2022 (PMC9402343; doi:10.1523/ENEURO.0052-22.2022)

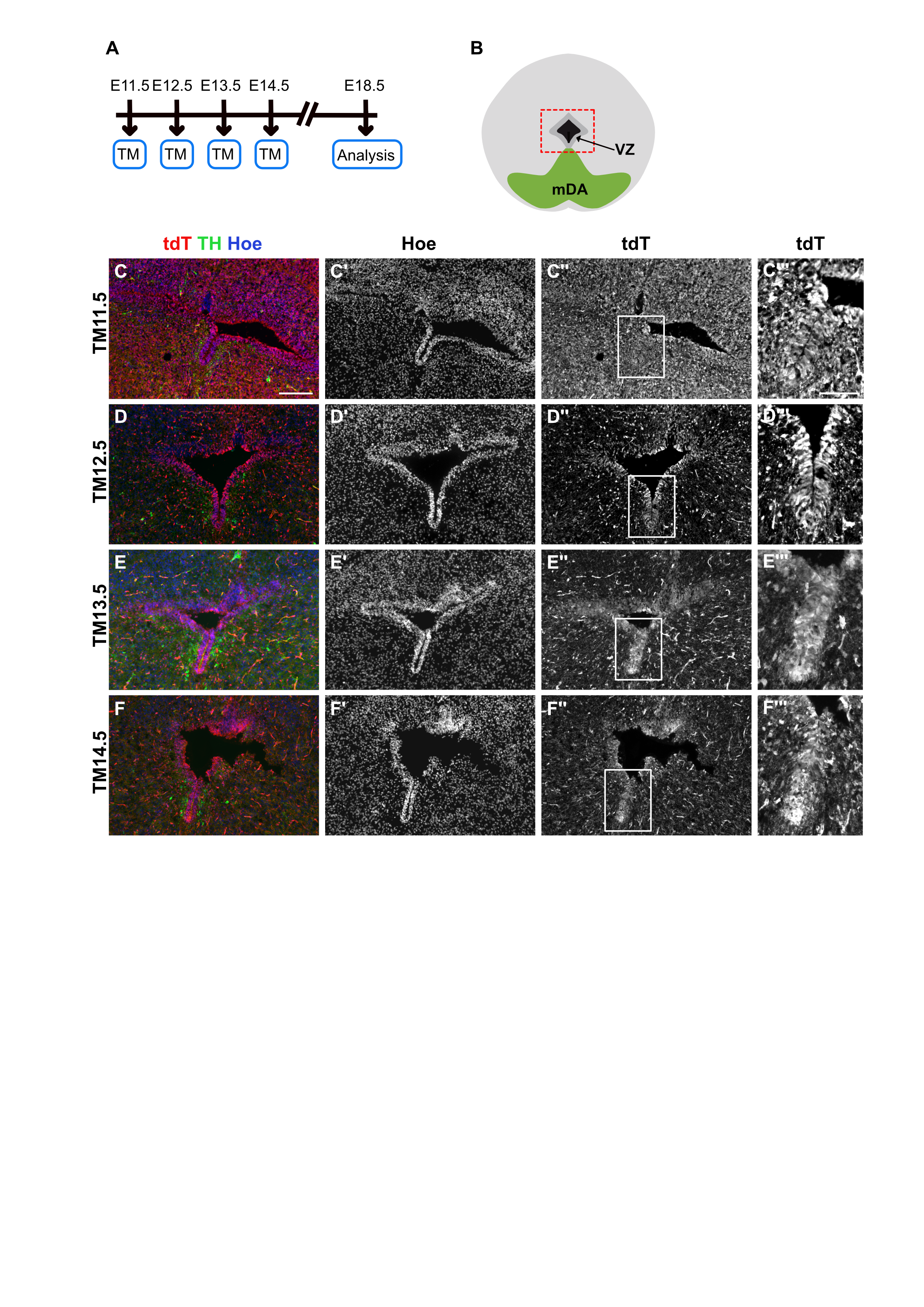

Supplement: Figure 2-1 — Cxcr4-inducible fate-mapping strategy results in labeling of cells in the midbrain ventricular zone between E11.5 and E14.5. A, Experimental timeline. B, Schematic of E18.5 ventral midbrain. The ventricular zone (VZ) and mDA neurons (green) are indicated. The dashed outline indicates the areas shown in C, D, E, and F. C, D, E, F, Immunostaining for tdT (red) and TH (green) on E18.5 coronal midbrain sections. Counterstain: Hoechst (Hoe). C′′′, D′′′, E′′′, F′′′, Areas indicated by the box in D′′, E′′, and F′′. Note that with TM11.5, almost all cells outside of the VZ express tdT; thus, the labeling of VZ cells is not as obvious as at subsequent time points of TM administration. Scale bars: C, C′′, E, E′′, F, F′′, G, G′′, 100 μm; C′′′, D′′′, E′′′, F′′′, 50 μm. Download Figure 2-1, TIF file. [file enu-eN-NWR-0052-22-s02.tif]

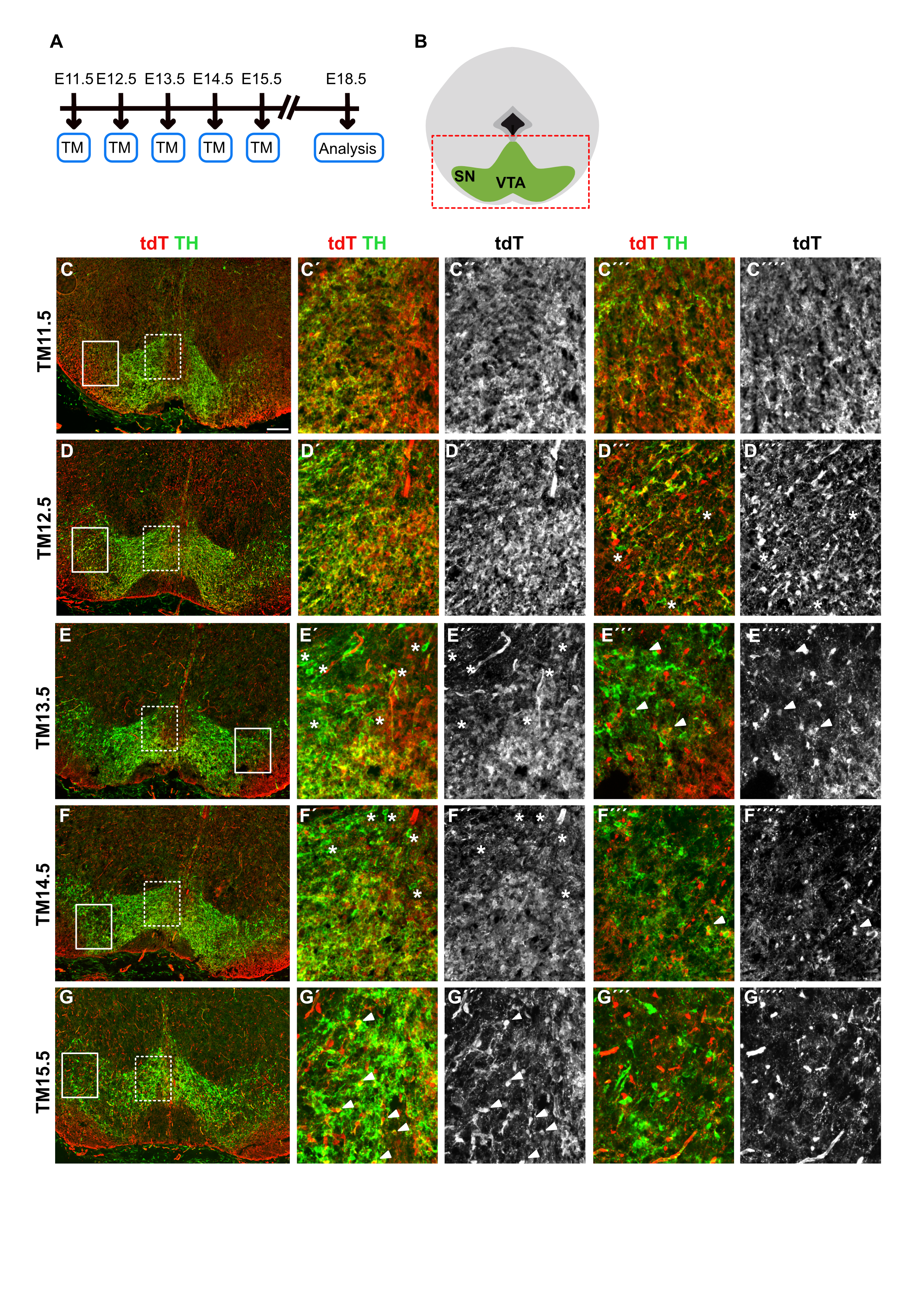

Supplement: Figure 3-1 — The Cxcr4 lineage contributes to mDA neurons between E11.5 and E15.5. A, Experimental timeline. B, Coronal view of the E18.5 brain. mDA neurons are indicated in green. The dashed outline indicates the area shown in C, D, E, F, and G. C, D, E, F, G, Immunostaining for tdT (red) and TH (green) on E18.5 coronal midbrain sections. C′–G′′, Area in the VTA indicated by the box (dashed line) in D, E, F, G, and H. C′′′–G′′′′, Area in the SNc indicated by the box (solid line) in D, E, and F. C–C′′′′, With TM11.5, almost all mDA neurons appear to be labeled with tdT. In addition, most cells in the ventral midbrain appear to be positive for tdT. D–D′′′′, With TM12.5, few mDA neurons do not coexpress tdT (indicated by asterisks). E–F′′′′, With TM13.5 and TM14.5, many tdT-negative mDA neurons (indicated by asterisks) are detected in the dorsal part of the VTA. In the SNc, only a few double-labeled mDA neurons (indicated by arrowheads) are present. G–G′′′′, With TM15.5, only a few tdT-expressing mDA neurons (indicated by arrowheads) are present in the VTA, and double-labeled cells are essentially absent from the SNc. Scale bars: C, D, E, F, G, 100 μm. Download Figure 3-1, TIF file. [file enu-eN-NWR-0052-22-s03.tif]

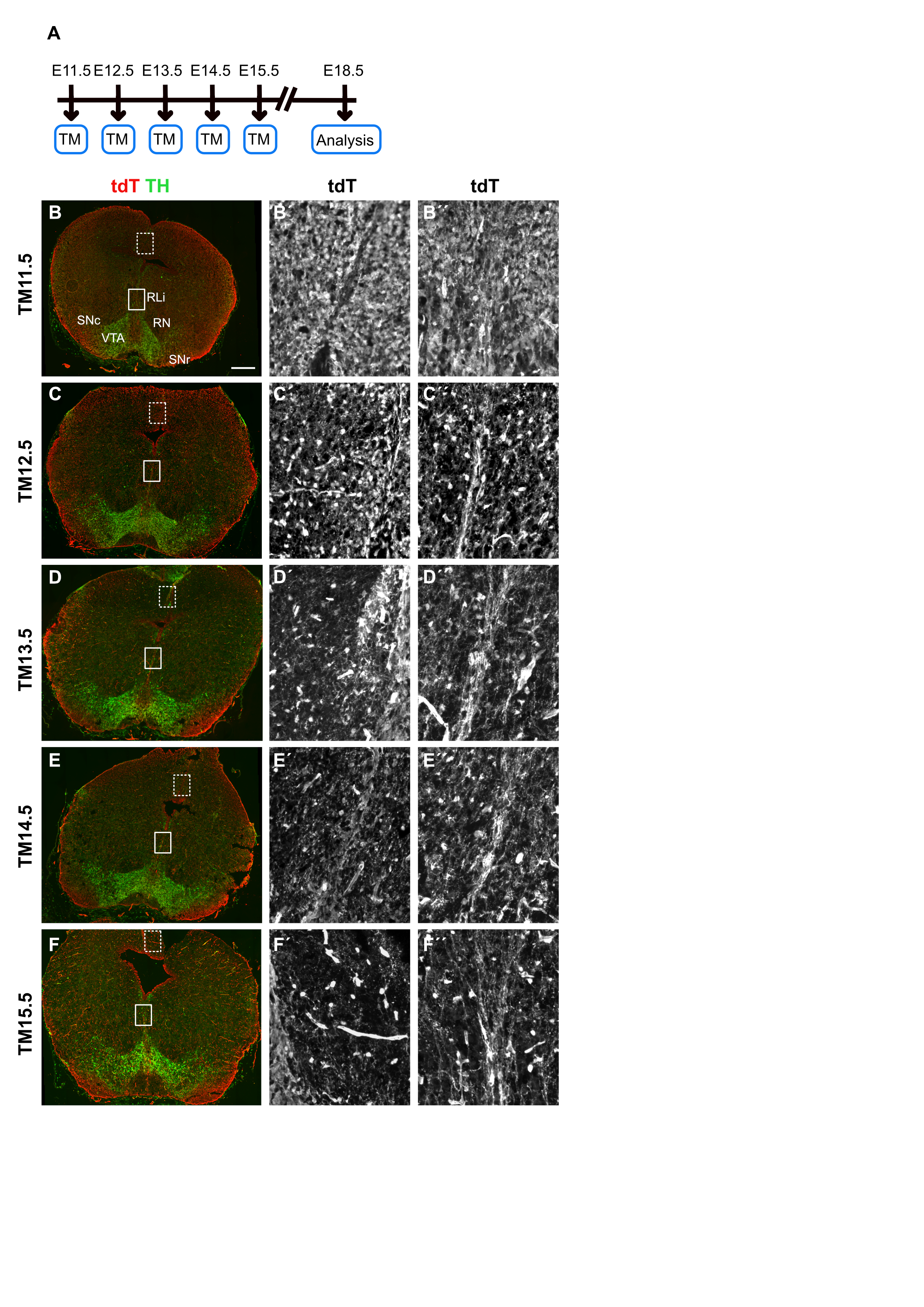

Supplement: Figure 3-2 — The Cxcr4 lineage contributes cells to the entire midbrain between E11.5 and E15.5. Analysis at E18.5. A, Experimental timeline. B, C, D, E, F, Immunostaining for tdT (red) and TH (green) on E18.5 coronal midbrain sections. Rli, Rostral linear nucleus; RN, red nucleus. B′, C′, D′, E′, F′, Area in the dorsal midbrain indicated by the box (dashed line) in C, D, E, F, and G. B′′, C′′, D′′, E′′, F′′, Area including the RLi in the ventral midbrain indicated by the box (solid line) in B, C, D, E, and F. B–B′′, With TM11.5, almost all cells in the selected areas appear to express tdT. B–F′′, The number of tdT-expressing cells decreases progressively from TM11.5 to TM15.5. Scale bar, 200 μm. Download Figure 3-2, TIF file. [file enu-eN-NWR-0052-22-s04.tif]

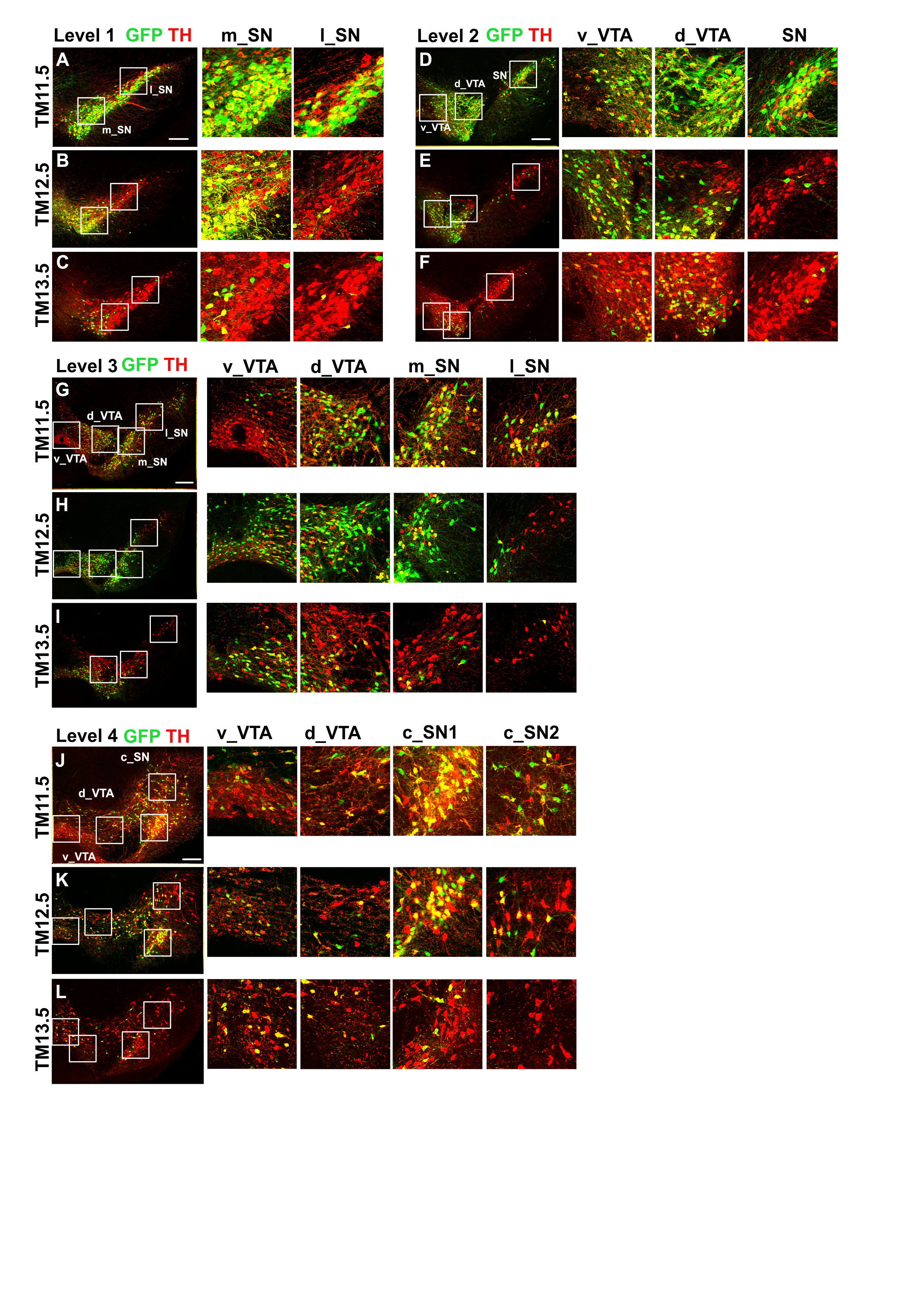

Supplement: Figure 4-1 — The Cxcr4 lineage contributes to progressively more restricted populations of mDA neurons over the course of development. A–L, Immunostaining for tdT (red) and TH (green) on P30 coronal midbrain sections: medial SNc (m_SN), lateral SNc (l_SN), dorsal VTA (d_VTA), ventral VTA (v_VTA), and caudal SNc (c_SN). Note that the overview images (A–L, left panels) are the images that are also shown in Figure 4. Boxes indicate the higher-magnification areas shown to the right. Scale bar, 200 μm. Download Figure 4-1, TIF file. [file enu-eN-NWR-0052-22-s05.tif]

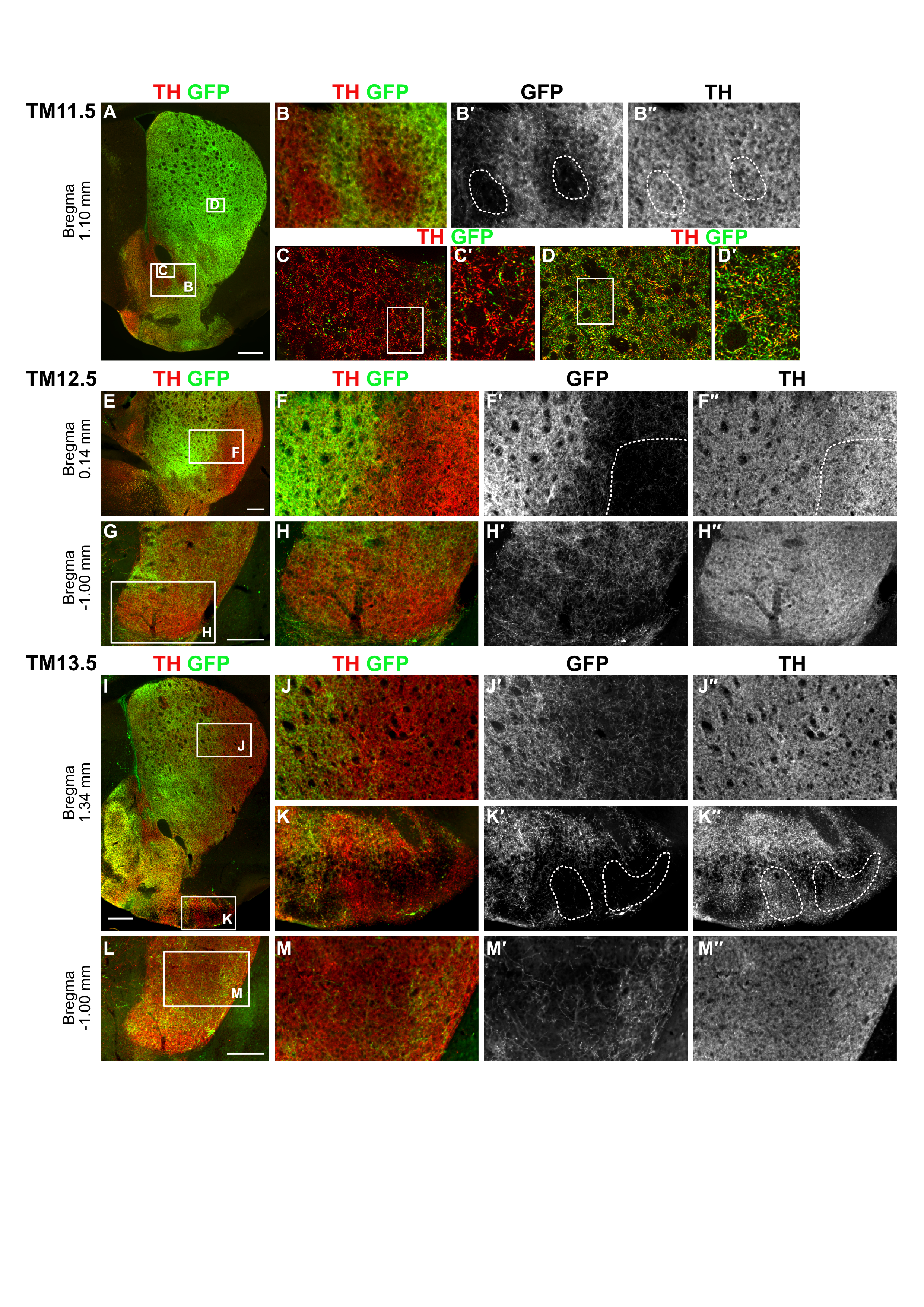

Supplement: Figure 5-1 — Innervation of projection targets by mDA neurons of the Cxcr4-lineage. (A–K′′) Immunostaining for tdT (red) and TH (green) on P30 coronal sections. Areas outlined by dashed lines indicate regions that are essentially devoid of GFP-positive axons (B′,B′′, F′,F′′, K′,K′′). Note that these areas are surrounded by regions with low density GFP-positive innervation. In these low density innervation areas, GFP- and GFP+ axons are intermingled (C, C′). In areas with the highest density of GFP-positive fibers, the large majority of fibers appear to be GFP-positive (D, D′, F). (E,G) Higher magnification of selected areas in the overview images shown in Figure 5 (E corresponds to Figure 5L, G to Figure 5R). Scale bar: 500 μm. Download Figure 5-1, TIF file. [file enu-eN-NWR-0052-22-s06.tif]
